# Compounding Risks Caused by Heat Exposure and COVID-19 in New York City: A Review of Policies, Tools, and Pilot Survey Results

**DOI:** 10.1142/S2345737621500159

**Published:** 2021-09-22

**Authors:** Jennifer Bock, Palak Srivastava, Sonal Jessel, Jacqueline M. Klopp, Robbie M. Parks

**Affiliations:** The Earth Institute, Columbia University New York, NY, USA; Stuyvesant High School, New York, NY, USA; WE ACT for Environmental Justice, New York, NY, USA; The Earth Institute, Columbia University New York, NY, USA; The Earth Institute, Columbia University New York, NY, USA; Mailman School of Public Health Columbia University, New York, NY, USA

**Keywords:** Social vulnerability, health inequity, heat exposure, COVID-19, New York City, environmental justice, climate justice, social justice

## Abstract

The Coronavirus Disease 2019 (COVID-19) pandemic changed many social, economic, environmental, and healthcare determinants of health in New York City (NYC) and worldwide. COVID-19 potentially heightened the risk of heat-related health impacts in NYC, particularly on the most vulnerable communities, who often lack equitable access to adequate cooling mechanisms such as air conditioning (AC) and good quality green space. Here, we review some of the policies and tools which have been developed to reduce vulnerability to heat in NYC. We then present results from an online pilot survey of members of the environmental justice organization WE ACT for Environmental Justice (WE ACT) between July 11 and August 8, 2020, which asked questions to evaluate how those in Northern Manhattan coped with elevated summer heat in the midst of the COVID-19 pandemic. We also make some policy recommendations based on our initial findings. Results of our pilot survey suggest that people stayed indoors more due to COVID-19 and relied more on AC units to stay cool. Survey responses also indicated that some avoided visiting green spaces due to concerns around overcrowding and did not regularly frequent them due to the distance from their homes. The responses also demonstrate a potential racial disparity in AC access; AC ownership and access was highest amongst white and lowest amongst Latino/a/x and Black respondents. The impacts of COVID-19 have highlighted the need to accelerate efforts to improve preparedness for extreme heat like the City of New York’s AC and cooling center programs, heat ventilation and air conditioning (HVAC) retrofitting, equitable green space expansion, and stronger environmental justice community networks and feedback mechanisms to hear from affected residents. Conducting a survey of this kind annually may provide an additional effective component of evaluating cooling initiatives in NYC.

## 1. Introduction

Severe acute respiratory syndrome coronavirus 2 (SARS-CoV-2) virus, which causes COVID-19, has resulted in over 4.4 million deaths worldwide. New York City (NYC) was one of the earliest and worst hit cities in the United States, with over 34,000 confirmed deaths to date (DOHMH 2021). In the summer of 2020, COVID-19 potentially heightened the risk of heat-related health impacts in NYC, in large part because people were encouraged to stay at home more to isolate from potential transmission of COVID-19. In Summer 2020, New Yorkers experienced several multi-day episodes of elevated heat (Davitt 2020). COVID-19 and heat exposure act as compounding health risks which, when combined with social and structural processes such as systemic racism, can make low-income communities of color more socially vulnerable and further perpetuate inequitable health outcomes.

The built environment plays a major role in exacerbating the impacts of extreme heat events. In particular, the urban heat island (UHI) effect exacerbates the impacts felt by extreme heat events and makes cities such as NYC particularly at high risk of adverse health outcomes. The UHI effect is the phenomenon in which urban environments experience higher surface and air temperatures than rural and suburban areas due to a higher density of buildings and impervious surfaces that radiate heat, a lack of green infrastructure that facilitates evaporative cooling, and heat exhaust from heavy vehicle traffic and other human activities (Rosenzweig *et al*. 2006; Hart and Sailor 2009). The UHI effect can make the nighttime air temperature of a large city 2–5°F (1–3°C) hotter than rural and suburban areas and as much as 22°F (12°C) warmer at night (Oke 1997; US EPA 2008, 2014). Surface temperatures tend to be hotter during the day due to surfaces such as pavement and buildings heating up from solar absorption (US EPA 2008). Surface temperatures and air temperatures vary depending on differences in land use type. For example, the air temperature tends to be cooler near surfaces such as parks and vegetated areas due to evaporative cooling and hotter near dense areas. Built-up areas, with concrete, metal or asphalt surfaces, more efficiently absorb and retain heat than green areas, which makes built-up areas retain heat for longer leading to the UHI effect. UHI effects are not equally felt across the city. Neighborhoods with less vegetation, and more industrial spaces experience higher temperatures (Rosenzweig *et al*. 2006). Often, these areas are predominantly neighborhoods of color and low-income (Madrigano *et al*. 2015; Casey *et al*. 2017; Vant-Hull *et al*. 2018). Air temperatures tend to take longer to cool down at night due to the UHI effect. When combined with elevated temperatures due to heat exposure, potentially fatal health risks emerge for residents living in poorly ventilated buildings surrounded by industrial, built-up areas that absorb a lot of heat (Quinn *et al*. 2014, 2017).

High temperatures, caused by extreme heat and exacerbated by the UHI effect, can lead to health outcomes including hospitalization from cardiovascular and respiratory diseases. Direct heat-ascribed health impacts include heat stress, dehydration, dizziness, fainting, heat stroke, and death (O’Neill and Ebi 2009). Many other deaths are attributable to deviations of temperature from long-term averages (Parks *et al*. 2020; Gasparrini *et al*. 2015). Heat exposure can also cause behavioral and mental health problems related to the stress of coping with elevated heat, leading to increases in deaths from assault and suicide (Burke *et al*. 2018; Parks *et al*. 2020). The underlying factors that make low-income communities of color more socially vulnerable to climate hazards such as extreme heat are the same factors that are causing inequitable health outcomes in the COVID-19 pandemic. COVID-19 and extreme heat together create cascading health risks for socially vulnerable populations (Thomas *et al*. 2020). High heat exposure combined with pre-existing health conditions and lack of access to cooling mechanisms such as air conditioning can further increase the severity of COVID-19 symptoms and make the disease more deadly. The COVID-19 pandemic has also made it harder for governments and community organizations to implement heat-health action plans and reach socially vulnerable populations which further increases heat-related health risks in these communities (Martinez *et al*. 2020).

Government and community institutions play a key role in addressing health inequities that are exacerbated by the compounding risks of extreme heat and COVID-19. Various policies and data tools have been introduced by the City and State in New York to address the health impacts caused by extreme heat. These include the Home Energy Assistance program (HEAP), NYC Cool Roofs, Cool Neighborhoods NYC, Cooling Centers, Cool It NYC, the Heat Vulnerability Index (HVI), Get Cool NYC, and the Be a Buddy pilot program.

Our aims were to (i) provide an initial overview of how effective NYC government’s interventions have been in targeting those most vulnerable to heat-related health issues, particularly during the COVID-19 pandemic; (ii) share the findings of a survey we conducted with the environmental justice organization WE ACT during Summer 2020; and (iii) share recommendations for how to improve the City’s cooling policies to ensure NYC’s most vulnerable communities are protected given the compounding risks they face from extreme heat and COVID-19.

### 1.1. Overview of heat exposure and vulnerability in NYC

The City of New York started to address heat-related public health impacts in 2009 through the planning process of the NYC Climate Adaptation Task Force. The City addressed excessive heat events through emergency planning and preventive programs implemented by the NYC Department of Health and Mental Hygiene (DOHMH) (Rosenthal *et al*. 2014). Since 2009, many policies and interventions such as opening cooling centers and expanding green space have emerged as responses to the problem of heat-related vulnerability. In order to implement these interventions effectively and equitably, it is important to have a clear sociological, epidemiologic, spatial, and ecological understanding of how heat exposure poses disproportionate health risks for disadvantaged communities.

Beyond the UHI effect, structural social factors in NYC have contributed to elevated temperature exposure in disadvantaged communities. Historical policies, such as redlining and racist zoning practices, led to widespread disinvestment in infrastructure, including green space and high quality housing in low-income communities of color. Neighborhoods such as the South Bronx and Harlem are considered “micro urban heat islands” and have high levels of heat exposure (Hamstead and WE ACT 2016; Hoffman *et al*. 2020). Formerly redlined areas can have land surface temperatures as much as 12°F (7°C) hotter than non-redlined areas (Hamstead and WE ACT 2016; Hoffman *et al*. 2020). The South Bronx, and Harlem in Northern Manhattan experience higher temperatures relative to the rest of NYC because of the large amount of heat-trapping materials such as concrete and asphalt and lack of cooling mechanisms such as green space (Hamstead and WE ACT 2016; Vant-Hull *et al*. 2018; Aram *et al*. 2019).

Social vulnerability to physical and health hazards such as extreme heat and COVID-19 is driven by physical and socioeconomic factors. Heat vulnerability is a function of physical vulnerability and social vulnerability. Low-income communities of color experience higher heat vulnerability due to physical factors such as the UHI effect and various socioeconomic factors including income, and access to cooling mechanisms that make it more difficult to cope with extreme heat (Rosenthal *et al*. 2014). Between 2000 and 2012, Black people were estimated to account for 50% of heat-related deaths in NYC (DOHMH *et al*. 2014) even though they only make up 25% of the population (US Census Bureau 2019). Within NYC, the Harlem community is particularly prone to heat-related health impacts. Due to residential buildings being lower and more widely spaced, a lack of surface winds, and warm southerly winds that pick up heat as they move up Manhattan, apartments and the urban canyons surrounding them absorb more sunlight and thus trap in more heat, making Harlem one of the warmest neighborhoods in NYC (Vant-Hull *et al*. 2018; Rosenzweig *et al*. 2006; Hamstead *et al*. 2016). The City’s HVI map confirms the high heat vulnerability Harlem residents experience showing various Central and East Harlem neighborhoods with high heat vulnerability scores of 4 or 5 (which indicate the highest heat risk a neighborhood can experience) (DOHMH 2020).^1^ Socioeconomic factors such as a high percentage of residents on assisted living, the neighborhood being predominantly Black and the area having one of the lowest rates of air conditioning (AC) use in the city, are all reasons that contribute to Harlem experiencing locally elevated heat-related health impacts (Hamstead *et al*. 2016). Many of the same neighborhoods that experienced the highest number of COVID-19 cases so far are also neighborhoods that experience higher temperatures on average than the rest of NYC (WE ACT 2020; Cary Institute 2020; Harrington and Taub 2020; Brannan and Salamanca 2020). Comorbidities such as respiratory diseases, hypertension, diabetes, cardiovascular disease, that make people more vulnerable to dying from COVID-19, are the same underlying health conditions that make people more vulnerable to dying from extreme heat (Martinez *et al*. 2020). People with respiratory diseases are vulnerable to elevated heat, and they are likely more strongly affected by COVID-19 once infected. It is also the same social, economic, and political forces that have caused environmental injustices such as disproportionate levels of air pollution, industrial development, and lack of access to green space that make low-income communities of color more likely to develop underlying health conditions that increase their vulnerability to COVID-19 and extreme heat (Maantay 2007; Wu *et al.* 2020).

Black and low-income individuals 65 years old and older in NYC are less likely to have access to air conditioning (Rosenthal *et al*. 2014). The study found that overall heat-related death rates correlated with a prevalence of poor housing conditions, poverty, impervious land cover, high land surface temperatures, and lower AC access (Rosenthal *et al*. 2014). 85% of heat stroke deaths in NYC has been attributed to heat exposure at home and lack of air conditioning (CDC 2013; Walters *et al*. 2014; Madrigano *et al*. 2015; Vant-Hull *et al*. 2018). Studies of heat wave mortality support this trend, finding that the majority of fatal heat exposures occur inside the home (Madrigano *et al*. 2015; Fouillet *et al*. 2008; Wheeler *et al*. 2013; Walters *et al*. 2014).

Because most heat-related deaths occur from heat exposure in homes, there is a need to provide heat-vulnerable communities with indoor cooling mechanisms such as AC units. COVID-19 only increases the health risks of heat-vulnerable populations due to the compounding risks caused by underlying health conditions as well as the need to stay indoors for social distancing. In addition to unequal AC unit access, low-income households tend to be more at risk for heat illness due to the prohibitive costs of running AC. Two surveys conducted in NYC found the primary reason people do not own an AC is cost (Lane *et al*. 2014; Lee and Shaman 2017). In 2014, 94% of NYC households in low-poverty neighborhoods had access to a home AC unit compared with 82% in high-poverty neighborhoods. The percentage differences in AC ownership are even greater between the wealthiest and poorest areas of the city (MOR 2017). For many low-income households, who already have a high energy burden, using air conditioning to stay cool is not a viable option due to the increased electricity costs. On average, low-income households in New York spend 12.6% of annual income on energy while moderate-income households spend 6.4% of their income (NYSERDA 2017). According to a 2018 study, 15% of people reported never or rarely using their AC and 24% of people said they chose not to use their AC because of the cost (Lane *et al*. 2014; Madrigano *et al*. 2018). Utility bills can increase up to 30% during the summer due to the use of AC units (Spivack 2020). There is also a lack of awareness of local heat emergencies among heat-vulnerable populations. In a survey conducted during the summer of 2011, 30% of seniors surveyed were not aware of any heat warnings issued by the City and did not perceive themselves to be at risk and many did not identify air conditioning as an important health protection strategy (Lane *et al*. 2014). 34% of seniors did not own an AC or never/rarely used it because they either disliked AC (29%), did not feel hot (19%) or preferred using a fan (18%) (Lane *et al*. 2014).

Green space, such as urban canopies, irrigated green roofs, and cool roofs can mitigate the effects of the UHI effect (Tewari *et al*. 2019). Implementing green infrastructure practices can mitigate the negative environmental consequences that are associated with highly urbanized land cover (Altman 2013). Trees and vegetation reduce air and surface temperature by providing shade and evaporative cooling through evapotranspiration leading to potential temperature decreases of 6–9°F (3.3–5°C) (US EPA 2014). Planting trees around houses and apartments can increase cool energy savings by 47% (Urban Design Forum 2020). Parks, especially parks with a high tree density, are also very effective outdoor cooling spaces. However, due to a legacy of racist urban planning practices, there is an inequitable distribution of parks which contributes to the increased UHI effect experienced in neighborhoods of color. Policies such as redlining, a federal housing policy started in the 1930s that marked communities of color as “undesirable” for home loans, have caused a legacy of disinvestment and a lack of green infrastructure in communities of color (Nardone *et al*. 2021). Surveying 37 cities, a study found that areas graded D, which were primarily racial and ethnic minority communities, have 23% tree canopy cover today compared to grade A areas that were predominantly white communities which have 43% tree canopy cover today (Locke *et al*. 2020). Another study that analyzed redlining and heat maps in over 100 cities in the U.S. confirms the inequitable outcomes of redlining, finding that redlining can be directly linked to higher levels of heat exposure in low-income communities of color. For example, formerly redlined areas in Richmond, VA can be as much as 5–15°F (2.8–8.3°C) hotter than wealthier, white neighborhoods due to lower levels of tree canopy cover (Hoffman *et al*. 2020; Plumer *et al*. 2020).

Some cities use heat vulnerability indices to make decisions about where to prioritize heat interventions. In NYC, researchers developed a HVI which was later used by the City of New York (Madrigano *et al*. 2015). The HVI study incorporated environmental factors such as building density, land use, proportion of area covered by trees or vegetation and estimated spatial variability in temperature using daytime surface temperature and overnight air temperature. The study found that those most vulnerable to a heat wave were Black individuals 65 years old and older, and individuals who received public assistance. Deaths during a heat wave were more likely to occur at home rather than at a hospital or other institutions possibly due to social isolation, lack of mobility, and lower AC access. Heat wave-related deaths were more likely to occur in areas of the city with higher relative summer daytime surface temperatures and amongst people living in areas with poorer housing conditions such as buildings with deteriorating infrastructure and high rates of building violations and property tax delinquencies which can increase energy costs and create energy insecurity for residents (Rosenthal *et al*. 2014; Hernández 2016). They also found deaths were less likely among residents living in areas with more green space. Another study that developed a HVI for New York State (excluding NYC) found that urban areas with high housing density, less open space, and high proportions of elderly, minority populations, and lower-income households were the most vulnerable to heat (Nayak *et al*. 2018). The study consolidated 13 variables into four main drivers of vulnerability: social/language barriers, socioeconomic status, geography/location area, and elderly/social isolation. They found that within urban areas, language barriers and socioeconomic disadvantage caused the highest vulnerability to heat.

## 2. Methods

We conducted a review of NYC government’s programs, data tools and approaches to extreme heat, including government, not-for-profit, and community group reports in order to effectively evaluate the City’s response to mitigate the compounding risks vulnerable communities faced to heat exposure and COVID-19 during Summer 2020. We also conducted an online qualitative survey between July 11 and August 8, 2020 in partnership with the environmental justice group WE ACT for Environmental Justice (WE ACT). The survey aimed to understand how WE ACT members, primarily based in Northern Manhattan, were coping with heat in the midst of the COVID-19 pandemic. We shared the survey with WE ACT members through online channels including virtual events the organization held, text message alerts, and email reminders. The intention of working with WE ACT to conduct the survey was to build partnerships with community members through an existing community-based organization as part of an effort to conduct inclusive, community-based participatory research (CBPR). The CBPR method seeks to break down the hierarchies involved in traditional scientific research by involving community members through the research process (Israel *et al*. 1998). The collaboration was motivated by the urgent health risks posed by COVID-19 and extreme heat and the reality of the disproportionate number of Harlem residents affected or killed by these compounding threats. The goal of the research was to co-generate knowledge that would be beneficial for the community to develop their own solutions and improve social and policy outcomes. Partnering with WE ACT also provided a good spread in the sample surveyed because the organization’s membership represents a diverse set of demographics and has many members who are considered vulnerable to extreme heat due to factors such as age, race, chronic health conditions, and lack of AC access.

This study was approved by the Institutional Review Board at the Columbia Mailman School of Public Health.^2^

## 3. Government Programs and Tools

Heat exposure is becoming a more severe health risk because of climate change and it has been made an even more elevated health risk due to the COVID-19 pandemic. These heightened health risks and the inequitable impacts they cause make it worth reviewing the government’s policies to address heat-related health risks. Evaluating the government’s policies is key to understanding how the pandemic impacted their ability to protect communities most vulnerable to these multiplicative health risks. Here, we review New York State (NYS) and NYC government’s cooling programs; data tools to target heat-vulnerable communities; and relevant community group reports to analyze the effectiveness of these interventions. A reference of government agency terms can be found in Table 1, with a reference for NYC and NYS cooling programs and policies in Table 2.

### 3.1. State and local government programs

Various programs and regulations at the City and State level in New York aim to mitigate the impacts of elevated heat. City programs include NYC Cool Roofs, Cool Neighborhoods NYC, Cooling Centers, Cool It! NYC, Get Cool NYC, and the Be a Buddy pilot program. NYS also has the HEAP program. The City and State both have heat vulnerability indices that measure how vulnerable locations are to extreme heat, based on environmental and sociodemographic factors.

#### Cool Roofs NYC

Cool roofs reduce heat absorption and increase energy savings compared to other infrastructure measures such as green roofs or HVAC system retrofits (Urban Design Forum 2020). The Cool Roofs NYC program, launched in 2009, is a joint initiative between the Department of Buildings, NYC Small Business Services, NYC Service and the Mayor’s Office of Resiliency and Sustainability that provides job training and work experience in installing cool roofs. The program is funded by the City through programs such as Cool Neighborhoods NYC as well as supported by roof coating vendors, community groups and corporations that provide funding and in-kind assistance (Furman 2015). In 2011, the City updated a 2007 law to require 75% of the roof area on all new or substantially renovated roofs to be a highly reflective surface (Garodnick *et al*. 2011). Since the Cool Roofs NYC program started, more than 9.2 million square feet (854,708m^2^) of rooftops have been covered with a reflective coating (Kotecki 2018).

#### Green Roof Tax Abatement Law

In 2016, there were 736 green roofs installed of which the majority were in midtown and downtown Manhattan and few were in heat-vulnerable neighborhoods such as Northern Manhattan, Central Brooklyn, the South Bronx (Treglia 2018). The Green Roof Tax Abatement state policy was updated under the new New York State Green Roof Tax Abatement law that went into effect in July 2019 (DOF 2020). This legislation allows the City to offer higher tax abatements ($15 compared to $5.23 per square foot) to roofs in priority areas such as areas with sewage overflow and areas with little green space (Urban Design Forum 2020). The new law also requires high priority areas be selected based on environmental justice concerns as well as stormwater infrastructure and other environmental concerns (SWIM Coalition 2019). The Mayor’s Office of Sustainability will be responsible for determining the priority areas where green roofs should be built based on this criteria (SWIM Coalition 2020).

#### HEAP

NYS’s HEAP program, which is funded by the federal HEAP program that started in the 1970s, helps low-income New Yorkers pay for the cost of heating and cooling their homes. The program includes a regular HEAP benefit, emergency HEAP benefit, heating equipment repair or replacement benefit, clean and tune benefits, and a cooling assistance benefit. In 2013, Governor Cuomo originally allocated $366.8 million for the program. The HEAP program services about 80% of households that are eligible for Supplemental Nutrition Assistance Program (SNAP) benefits and about 20% of other low-income households (NYSERDA 2017). In the State of New York, most HEAP funding is used for heating assistance compared to cooling assistance. 65% of HEAP funding is allocated to heating assistance while only 2% is allocated for cooling assistance with the remainder used to fund crisis assistance, weatherization, and administration costs (MOR 2017; OTDA 2019; DOHMH 2020a). A new 2020–21 NYS HEAP plan will increase the cooling assistance allocation to 4% (OTDA 2020). Until reforms in 2017, HEAP cooling assistance funding was limited to only providing financial support to purchase and install air conditioning for low-income residents who had a documented medical need or qualified for certain public benefits such as SNAP. The program was further expanded under the 2020 Get Cool NYC program. National Grid now provides utility bill assistance that shows up as a monthly bill credit to low-income customers through the HEAP program (National Grid 2020). ConEdison customers who were eligible for HEAP also qualified for ConEdison utility bill assistance during Governor Cuomo’s state of emergency which was declared in March 2020 after the start of the pandemic (ConEdison 2020). Residents receiving federal housing subsidies are also now eligible for HEAP, when they were not before Summer 2020.

#### Cool Neighborhoods NYC

The Cool Neighborhoods NYC program, created in 2017 as part of the City’s larger OneNYC climate agenda, established the City’s first comprehensive heat resiliency program that focuses on reducing heat-related health impacts and deaths by lowering temperatures in heat-vulnerable neighborhoods and strengthening social networks. The $106 million program improved the City’s cooling centers as well as expanded the NYC Cool Roofs program and the HEAP program to allow qualifying households to apply for energy utility bill assistance related to air conditioning operation (MOR 2017).

#### Street Tree Planting

As part of the Cool Neighborhoods NYC program, the City also created a street tree planting initiative that targeted planting trees in heat-vulnerable areas identified based on the City’s HVI. The initiative committed $82 million to fund street tree planting in the South Bronx, Northern Manhattan, and Central Brooklyn which are neighborhoods identified to be disproportionately vulnerable to heat-health risks. The City also invested $16 million to plant trees in parks and $7 million to support forest restoration across all five boroughs (NYC Office of the Mayor 2020a).

#### Home Health Aide Training

The program also created climate risk training for home health aides. The City developed a partnership with three home care agencies to promote heat and climate-health information and engage home health aides as important participants in increasing climate resiliency. The initiative also includes using the home care agencies’ existing continuing education curriculum to educate 8,000 home health aides on climate risks and how to identify heat-related illnesses (NYC Office of the Mayor 2020a).

#### Be a Buddy Program

In 2018, as part of the Cool Neighborhoods NYC program, the Mayor’s Office of Resiliency and Department of Health and Mental Hygiene (DOHMH) created the Be a Buddy pilot program to promote community cohesion and collaboration with community groups to develop and test strategies for protecting at risk New Yorkers from the health impacts of extreme heat in heat-vulnerable areas including the South Bronx, Central Brooklyn, and Northern Manhattan (NYC Office of the Mayor 2020a). The POINT CDC, located in the South Bronx, is one of the community organizations that participated in the pilot program. The POINT CDC experienced success with the program, enrolling 100 people into its Be a Buddy network and reaching 500 people during a Summer 2019 extreme heat event (NYC-EJA 2020). The POINT CDC expanded the Be a Buddy program’s scope to help the Hunts Point community strengthen its preparedness to all types of climate-related extreme weather events. The Be a Buddy program currently has no dedicated funding source (NYC-EJA 2020).

#### Cool It! NYC

As part of the Cool Neighborhoods NYC program, the City created the Cool It! NYC plan to increase the amount of cooling amenities available to the public. The plan focuses on increasing the number of spray showers, drinking fountains, and trees in heat-vulnerable areas based on the City’s HVI. The Parks Department and New York City Housing Authority (NYCHA) turn on spray showers (sprinklers) when temperatures reach 80°F (26.66°C) or higher (NYCPARKS 2020).

#### Cooling Centers

Another major objective of the Cool Neighborhoods NYC program was to improve the cooling center program. The City opens designated cooling centers at locations such as community centers, senior centers, and public libraries during a heat emergency to provide a safe and cool place for heat-vulnerable community members to go (NYC OEM 2020; NYC311 2020a).

#### Get Cool NYC

The most recent program the City implemented is the Get Cool NYC program, which provided free air conditioners to low-income seniors who were enrolled in city benefit programs (NYC311 2020b). Get Cool NYC was created in May 2020 after it became clear that the City would not be able to open as many cooling centers and cooling amenities such as pools due to COVID-19 restrictions and heat-vulnerable populations would be more reliant on indoor cooling mechanisms such as air conditioning. Indoor cooling is one of the most effective measures to prevent heat-related health impacts due to the high rate of heat illnesses and deaths that occur from heat exposure at home (Madrigano *et al*. 2015; Fouillet *et al*. 2008; Wheeler *et al*. 2013). The City first started distributing free ACs for at-risk low-income seniors in the summer of 2008 through the federal HEAP program following an earlier pilot program (Rosenthal and Brechwald 2013). The increased need for indoor cooling mechanisms during COVID-19 helped push the City administration to expand their free AC program and create Get Cool NYC. Kizzy Charles-Guzman, the Deputy Director of the Mayor’s Office of Sustainability, had requested funding from the City to create a more comprehensive free AC program for years. At a public event, Ms. Charles-Guzman said COVID-19 is what made City officials take indoor heat vulnerability seriously enough to finally fund a free AC program (Charles-Guzman 2020a).

The Get Cool NYC program involved spending $55 million on 74,000 AC units for low-income seniors, including 22,000 for people in public housing. In June 2020, the program was updated after the City received approval from the New York State Public Service Commission to provide $70 million in financial assistance to vulnerable low-income New Yorkers to help subsidize their summer utility bills (NYC Office of the Mayor 2020b). This utility bill assistance program expands the State’s HEAP program and provides subsidies to people who qualify based on criteria such as having an underlying health condition, making below a certain income, and qualifying for city benefit programs such as SNAP benefits (NYC HRA 2020). The aid was estimated to provide cooling utility bill subsidies to 440,000 families providing up to $140 from June to October. A statement from the Mayor’s office read that officials reached out to over 180,000 low-income seniors on the topic and over 25,000 requested air conditioning units (Moloney 2020). On June 12, Mayor De Blasio’s Office released a statement saying, “eight times more air conditioners were installed in the apartments of low-income, vulnerable seniors in the first few weeks of the program compared to the number of installations for all of last year under the HEAP” (NYC Office of the Mayor 2020b).

Due to the ambitious timeline and scope of the program and the interagency involvement, there were various challenges in implementing the program. The original goal was to install 74,000 ACs by the end of July 2020, but by mid-August only 55,800 units were installed (Fitzsimmons 2020). As of July 2020, 10,000 AC units had been installed in NYCHA housing units (Culliton 2020). In November 2020, the Mayor’s office confirmed they installed more than 74,000 ACs (Charles-Guzman 2020b). The City contacted more than 450,000 households to identify eligible recipients through robocalls (Mercado 2020). Conducting outreach through robocalls may have led to fewer people following up on the call. Senior centers also helped conduct outreach through cold calls to register senior residents. Eventually, DOHMH partnered with volunteer community organizations to intake people. There were also administrative challenges with installing the ACs, including finding enough ACs and enough workers to install them. Initially, the National Guard was charged to help due to a labor shortage in the height of the COVID-19 pandemic when people were afraid to work (Charles-Guzman 2020b). The City also ended up needing to ask City employees to volunteer to install ACs. The program was managed by six City agencies: four agencies determined eligibility, Department of City Administrative Services (DCAS) bought ACs, and NYCHA and the Office of Emergency Management (OEM) shared oversight of installations. The Department of Aging, one of the agencies which help to identify eligible recipients, sent out an incorrect email saying the program had ended. There were also logistical issues with installing ACs in old buildings. Many apartments had window gates that prevented an AC from being installed. The City also had to find additional funding for the $55 million program after NYSERDA changed their mind about allocating $20 million to the program (Fitzsimmons 2020).

### 3.2. NYC data tools

The City has developed an array of data tools for its residents to learn about the causes and impacts of extreme heat and how to reduce their heat vulnerability including the HVI mapping tool, the Cool It! NYC mapping tool, and the Street Trees mapping tool. Table 3 provides a reference and description of the currently used tools.

The City adopted a HVI created in 2015 that measures how vulnerable neighborhoods are to heat-related health risks based on environmental variables including daytime summer surface temperature data, percent of green space, land surface temperature, and social variables including poverty measured by the percent of people living below the federal poverty level, percent of non-Latino/a/x Black residents data, and access to air conditioning data (Madrigano *et al*. 2015). Since its creation, the index has been updated to include data from 2018 (DOHMH 2020b).

**Figure 1.**
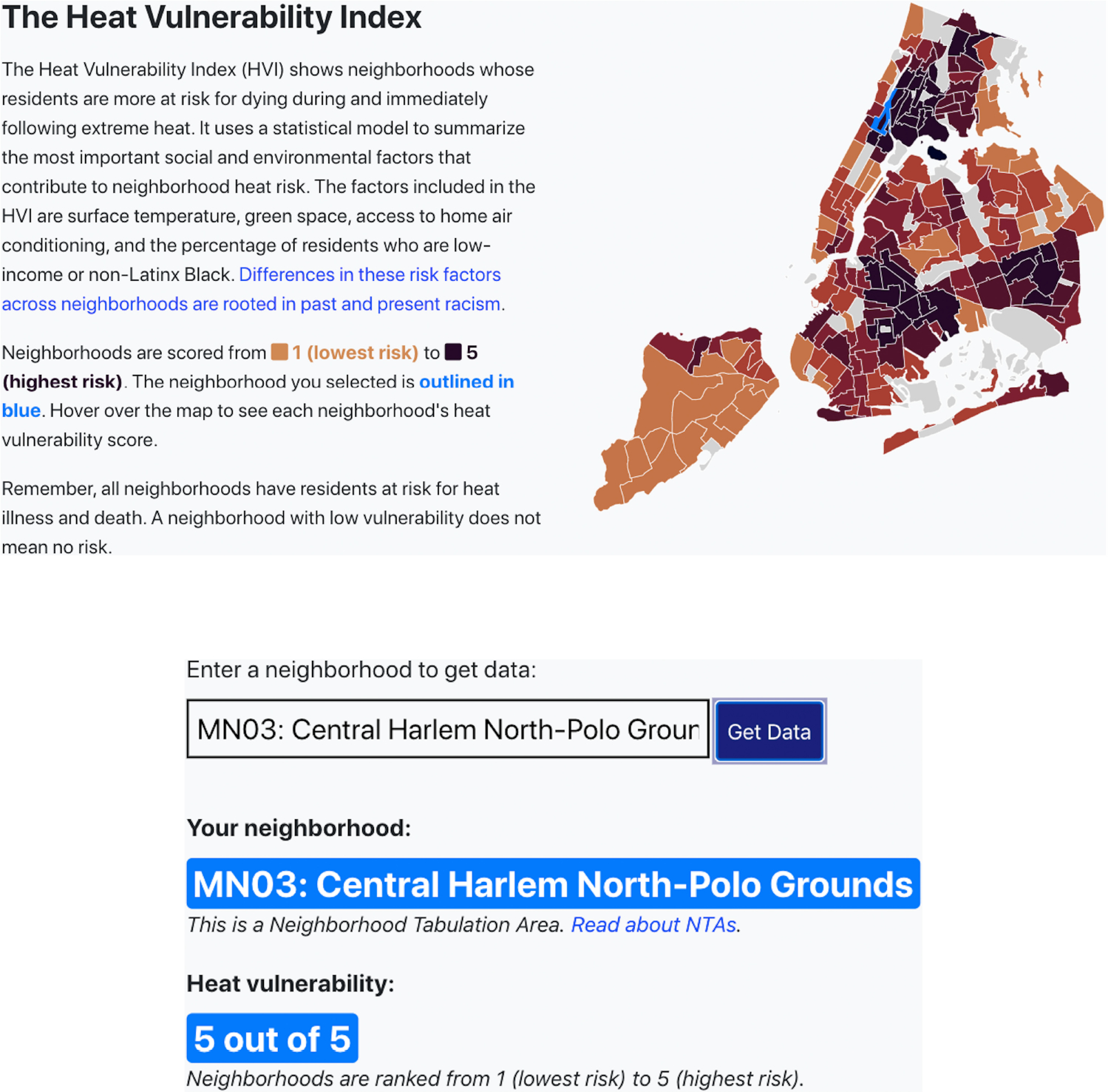
NYC Heat Vulnerability Neighborhood Map *Source*: DOHMH.

**Figure 2.**
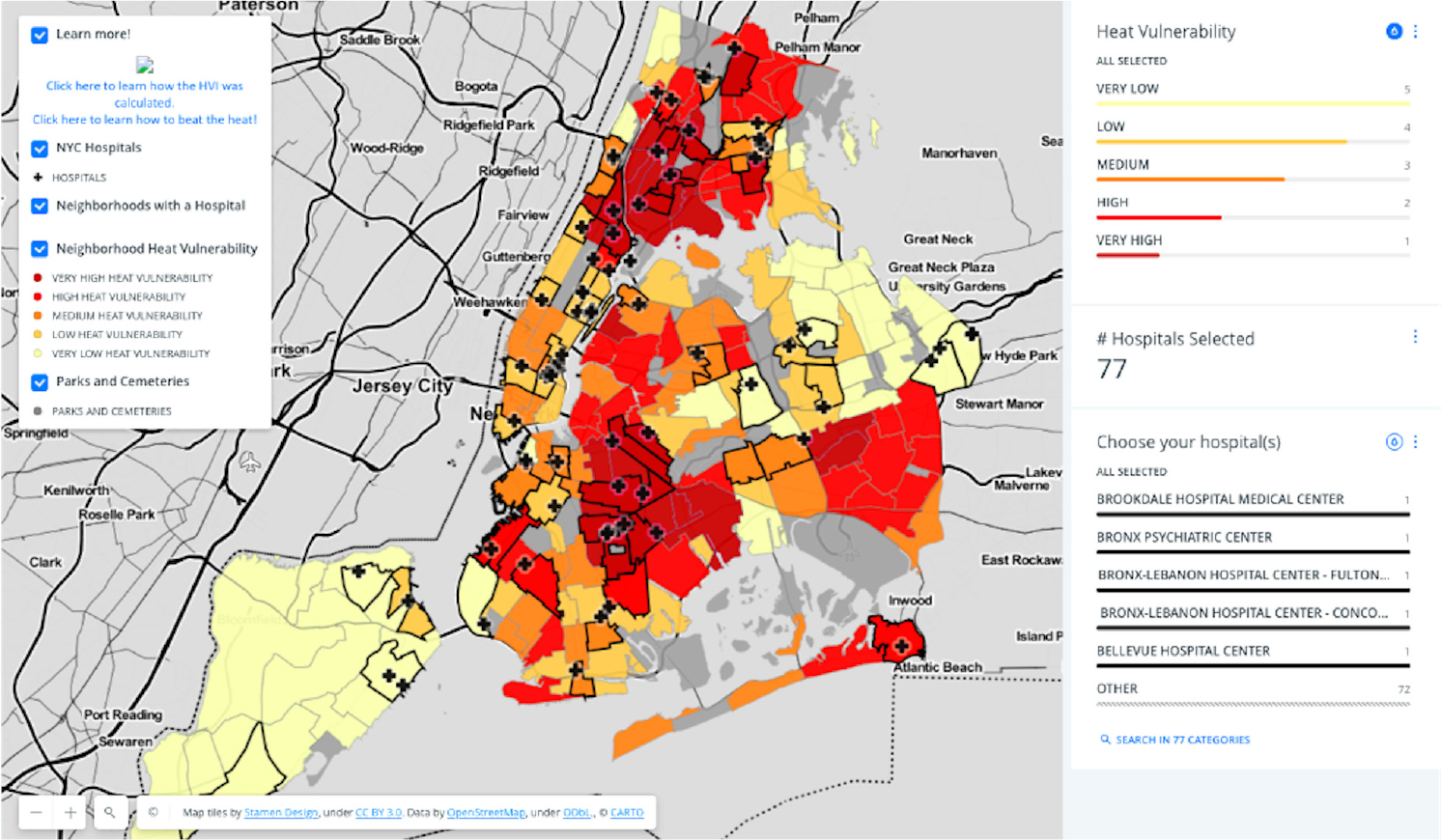
NYC HVI Map with Hospitals and Green Spaces *Source*: DOHMH.

The Department of Health has a HVI map that is broken down by neighborhood and allows users to look up their heat vulnerability by street address (DOHMH 2020c) (Figure 1). The Department of Health also has an HVI map with hospitals (DOHMH 2020d) (Figure 2). Additionally, this map allows the user to break down locations of hospitals based on the heat vulnerability of regions. However, the map groups parks and cemeteries together, which may be a misleading representation of green space that can be used as a cooling mechanism. The map also lacks addresses for or directions to the hospitals.

**Figure 3.**
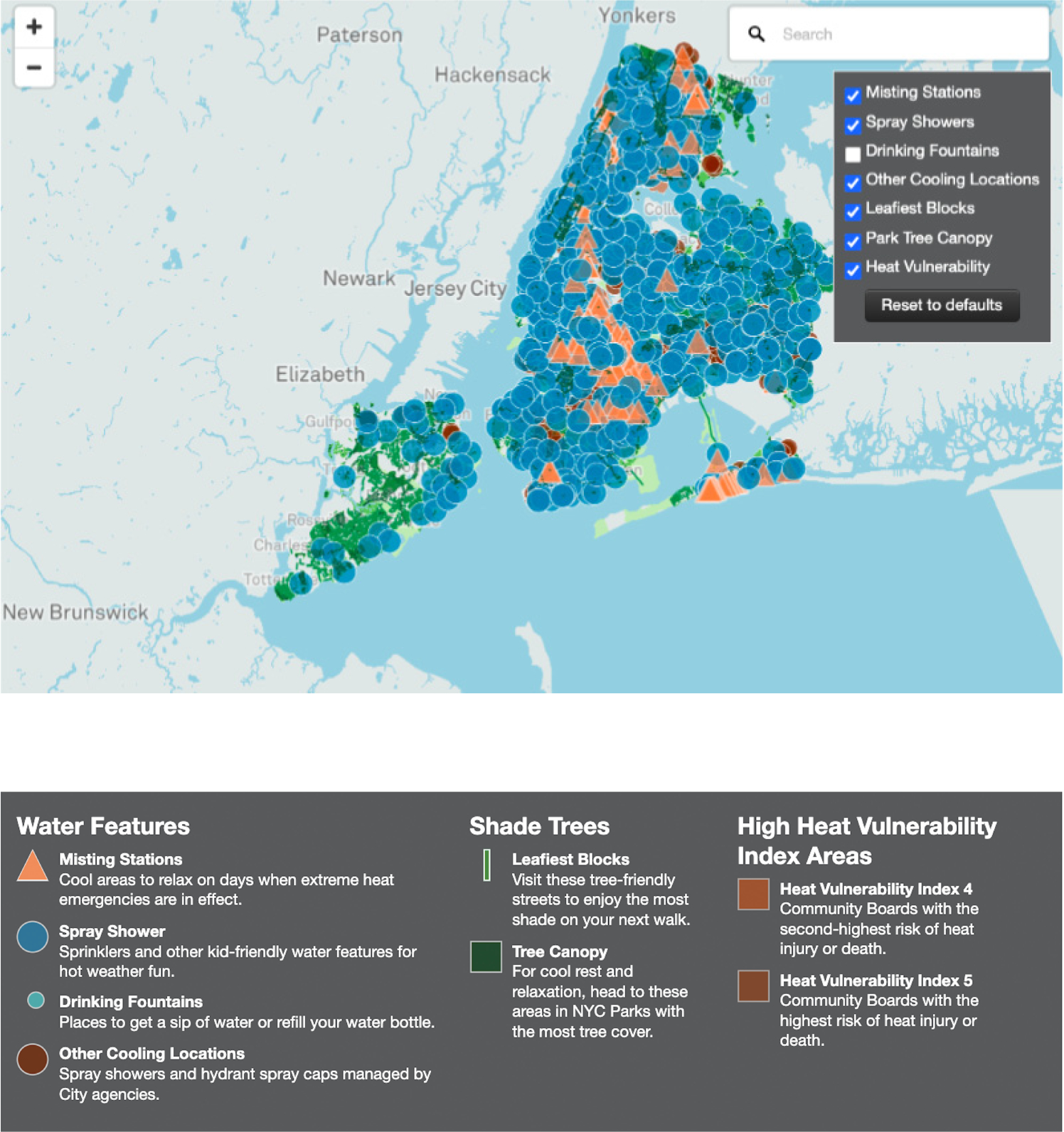
NYC Cool It! NYC Map *Source*: NYCPARKS.

The Cool It! NYC Map created by the NYC Parks Department shows a comprehensive list of cooling amenities communities can access to cool down during a heat emergency (Figure 3). The map shows locations of heat vulnerability, misting stations, spray showers, drinking fountains, leafiest blocks, and park tree canopy (NYCPARKS 2020). The map includes tags for amenities that are “only activated during a heat emergency”. The webpage does not define a heat emergency. The Parks Department also created a Street Trees Map which shows the locations of all street trees across NYC and includes information about the specific tree species. Both of these maps show urban tree canopy or location of street trees to show where trees are providing a cooling effect in the city.

## 4. Survey Results

The sample size of the survey was 206 respondents. 107 respondents answered a majority of questions and 26 respondents answered all the survey questions. The response rate for respondents who answered the majority of questions was 52% and the response rate for respondents who answered all survey questions was 13%. The survey completion rate may have been lower because there were some sensitive questions and it was optional to answer all questions. Some of the questions people consistently did not answer included questions about AC use in past summers, whether they felt safe from violence in green spaces and the influence of police on visits to green spaces. The majority of respondents were middle-aged or senior. 53 respondents were 16 to 49 years old and 30 respondents were 60 years old or older. The majority of respondents were Black or white: 24% of respondents identified as Black or African and 22% of respondents identified as white. 12% of respondents identified as Latino/a/x, Hispanic or of Spanish origin. 12% identified as mixed-race. 2% identified as Asian. The majority of respondents, approximately 77 people or 36% of respondents had a stable and high enough income to rent or own an apartment. 28 people or 20% of respondents lived in either transitional housing, Section 8 public housing, or a NYCHA apartment.

**Figure 4.**
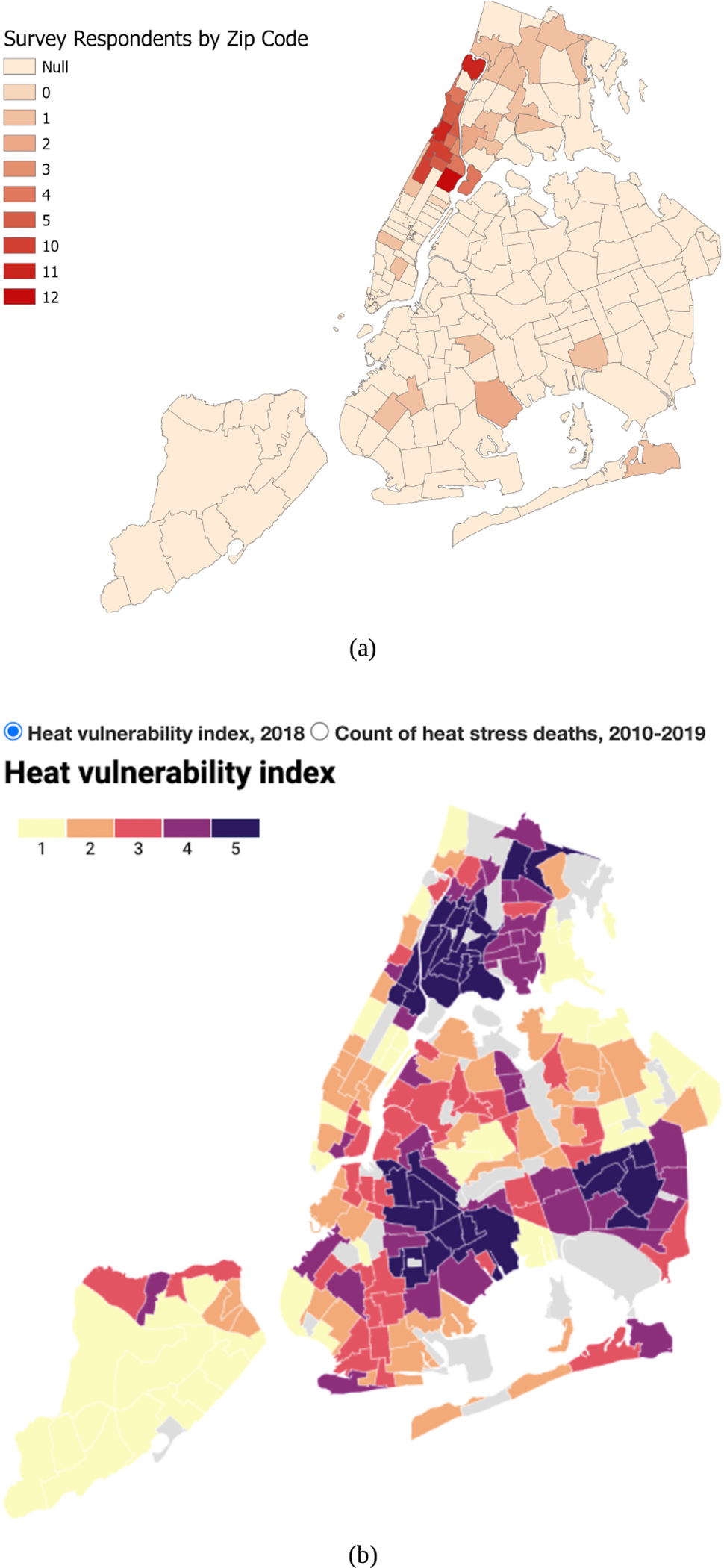
Map of (a) 2020 Extreme Heat COVID-19 Survey Results and (b) NYC Heat Stress HVI by Neighborhood Tabulation *Source*: DOHMH.

Based on the ZIP Code filled in during survey responses, the results show most survey respondents lived in Northern Manhattan with a small group living in Brooklyn, Queens, and the Bronx, as seen in Figure 4. 52 out of 111 respondents (47%) lived in areas with high heat vulnerability scores of either 4 or 5. 21 respondents in Northern Manhattan, 6 respondents in the Bronx, and 1 respondent in Brooklyn lived in ZIP Code areas with an HVI of 5. 12 respondents in Northern Manhattan, 3 respondents in Brooklyn, 4 respondents in the Bronx, and 2 respondents in Queens lived in ZIP Code areas with an HVI of 4.

**Figure 5.**
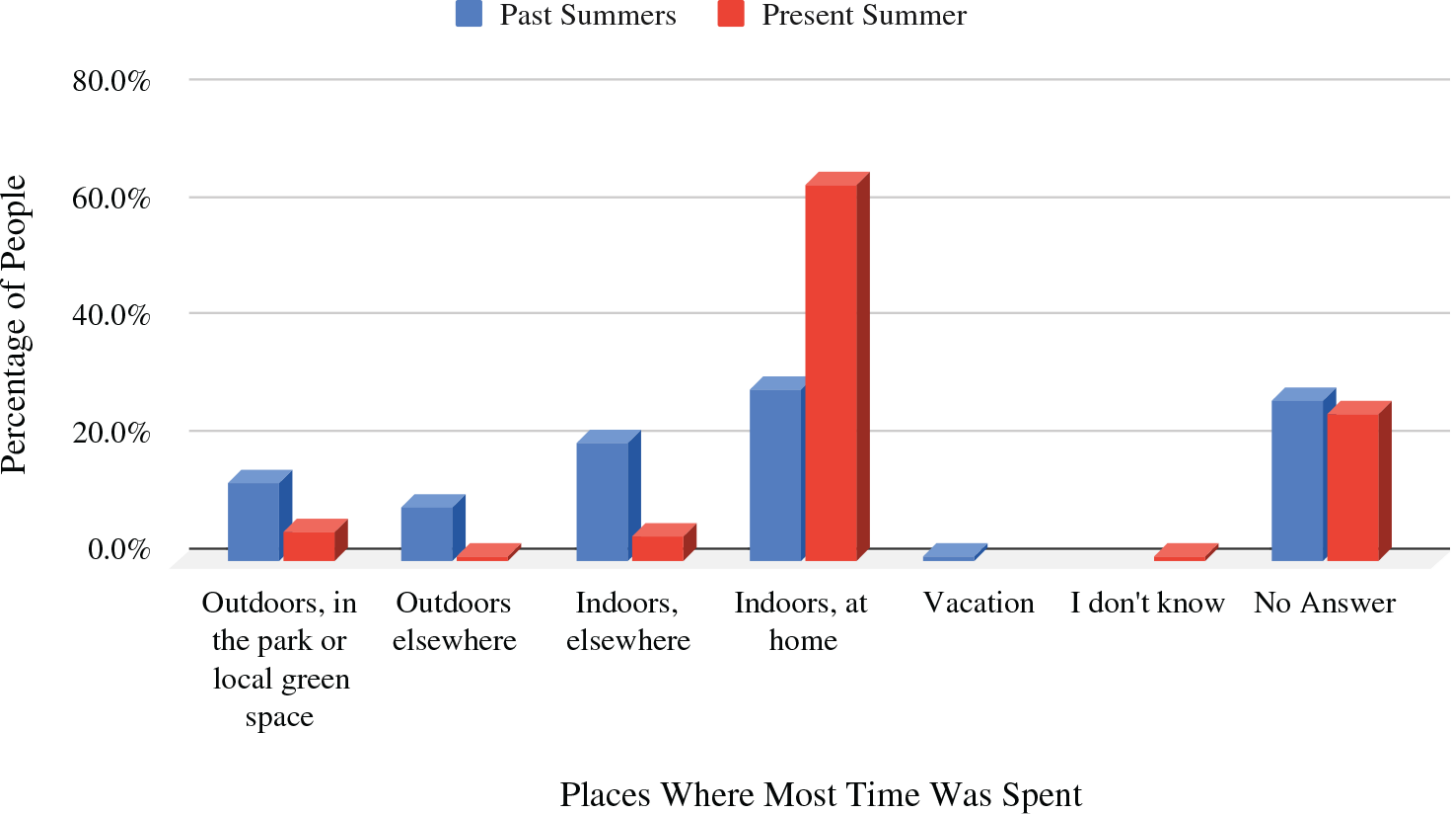
Comparison of How People Spent Most Time on Hot Days this Summer vs. Past Summers

**Figure 6.**
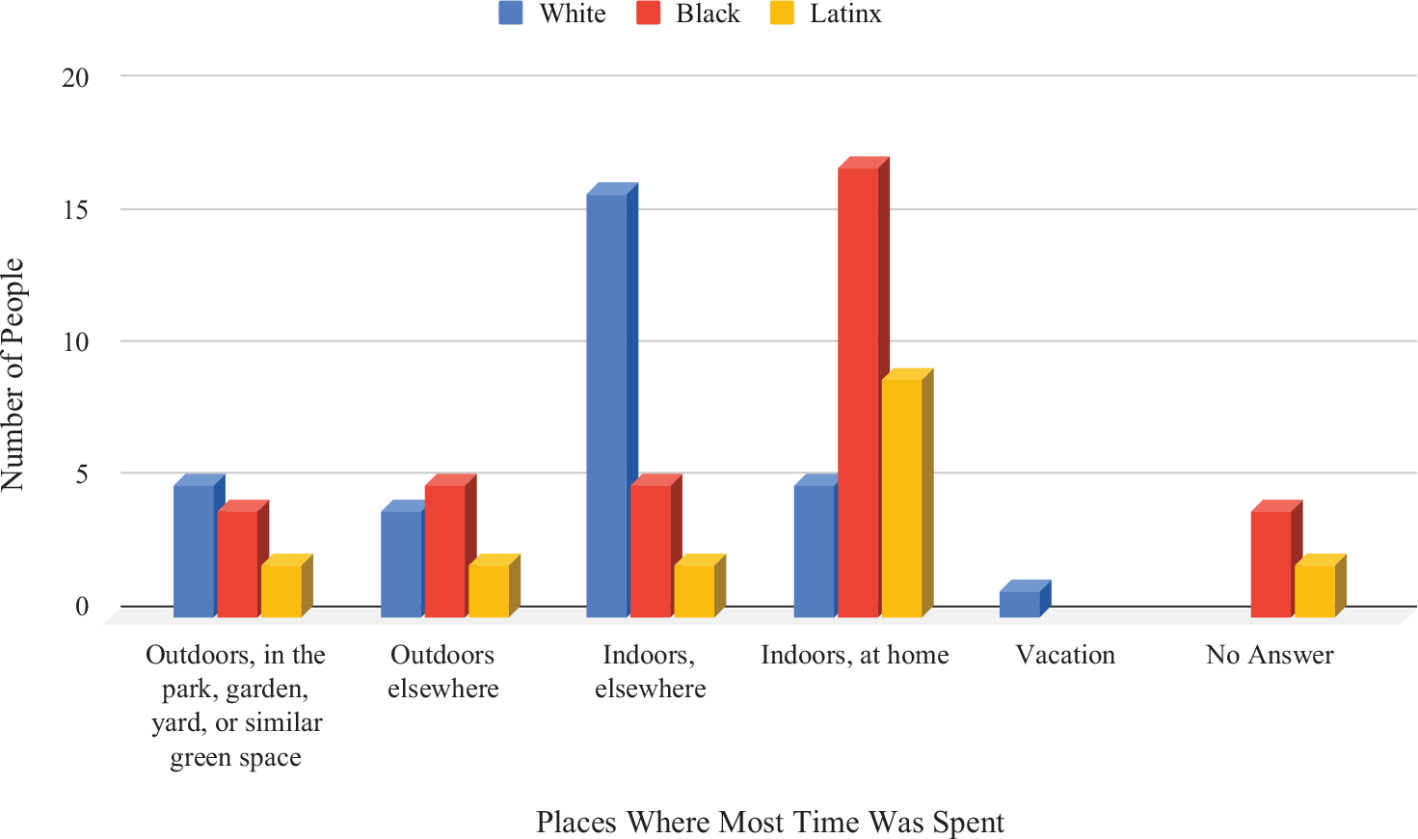
How People Spent Past Summers by Race

Survey results show that people spent less time outside in green spaces, outdoors, or elsewhere in Summer 2020 compared to past summers (Figure 5). In past summers, Black and Latino/a/x respondents spent more time indoors at home compared to white respondents who spent more time indoors (see Figure 6). The survey results show a large increase in people spending more time indoors in Summer 2020 compared to past summers. 92 people spent time indoors at home on hot days whereas in previous summers 42 people spent time indoors at home on hot days.

**Figure 7.**
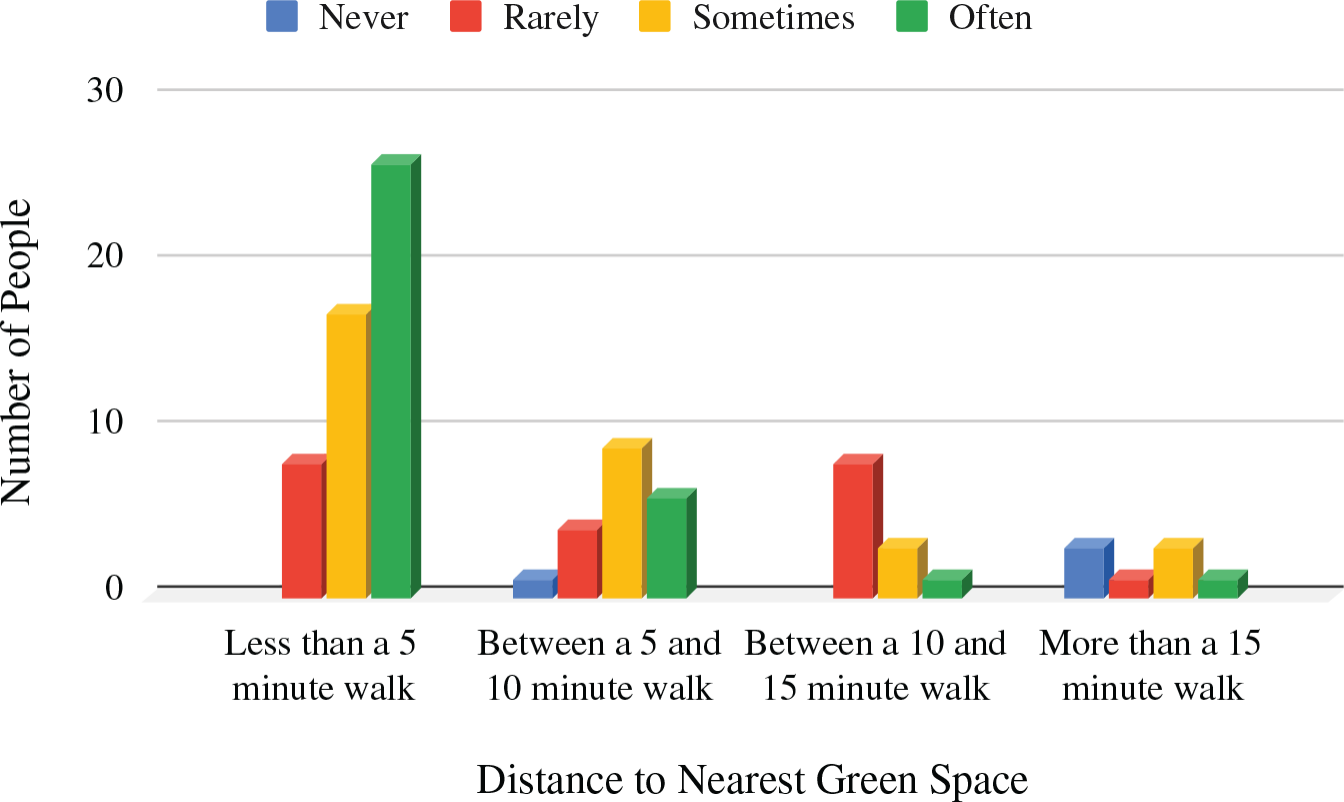
How Often People Visited Green Spaces Based on Distance to Them

**Figure 8.**
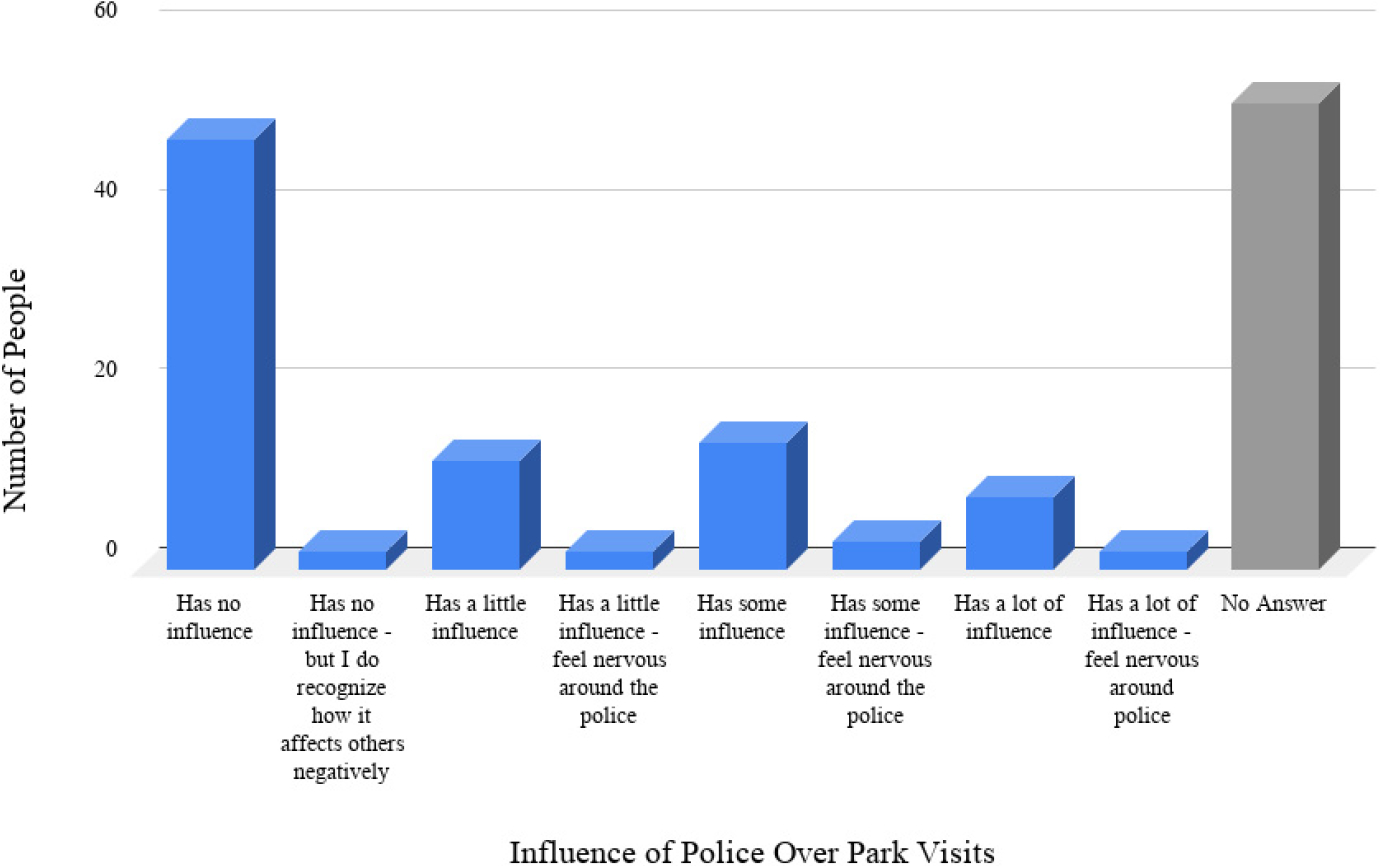
Breakdown of the Influence Police has Over Park Visits

Almost half of respondents (40%) see parks as a cooling mechanism. People spent less time in green spaces and outside than past summers. Our results also showed people who lived farther from green spaces visit green spaces less (Figure 7). According to survey comments, people expressed feeling unsafe in parks due to the concerns around parks being too small or crowded to properly maintain social distance. Respondent comments also confirmed survey results that distance to parks influenced how often they visit them. Others expressed concerns about going to parks due to fear of police presence. However, there is inconclusive evidence of the influence of police presence over people’s decision to visit parks or their experience at parks based on survey results (Figure 8).

### Comments about feeling unsafe in parks:

Quotations from the survey have been lightly edited to correct spelling and grammar:

“Often people aren’t wearing masks. The size of the green spaces is sometimes as big as my kitchen (which is small) so there’s not really space to relax. The park that is ‘nearby’ is about a mile away.”

“There are many people sitting in the green area at the same time and we cannot social distance.”

“The nearest green space has few benches and is mostly concrete. The more spacious park is about a 10-minute bus ride (or 30-minute) walk for me.”

“Sometimes the spaces are closed. Often people aren’t wearing masks.”

“I don’t go to any green space places and it seems that very few places that I go people don’t stay 6 Feet away.

“There too many without masks in the park. Many people are not complying. There is no enforcement of this requirement.”

“I recognize the extreme policing of this neighborhood and do see how it affects others negatively.”

“You never know the minds of an officer until you encounter them. I feel safer in the park with my granddaughter...”

**Figure 9.**
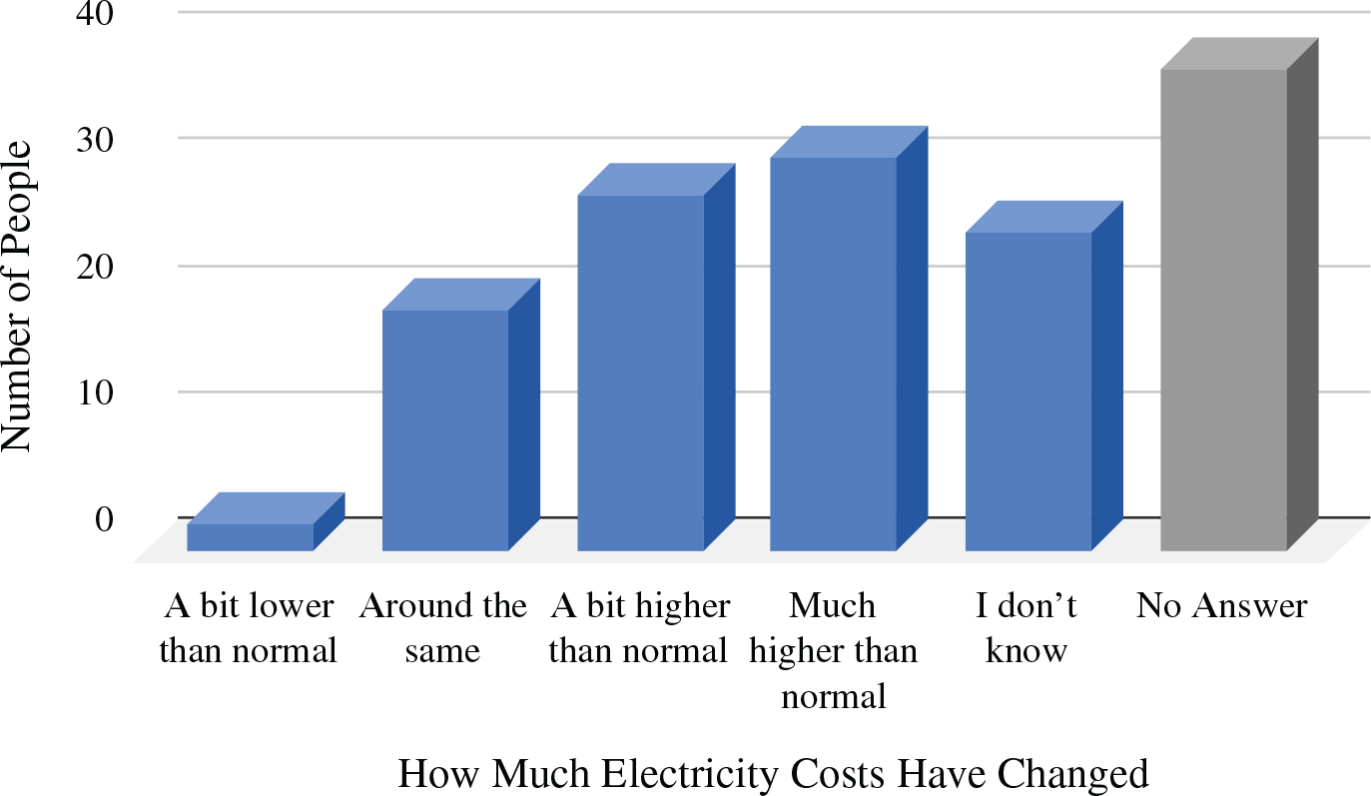
Breakdown of How Electricity Costs Have Changed Since Past Summers

At least 57.3% of survey respondents owned an AC. However, many did not answer this question. Electricity costs went up at least slightly for the majority of people most likely because more people stayed inside their homes (see Figure 9). Electricity costs went up at least slightly for 59 people. 28 people had electricity costs a bit higher than normal. 31 people had electricity costs much higher than normal.

**Figure 10.**
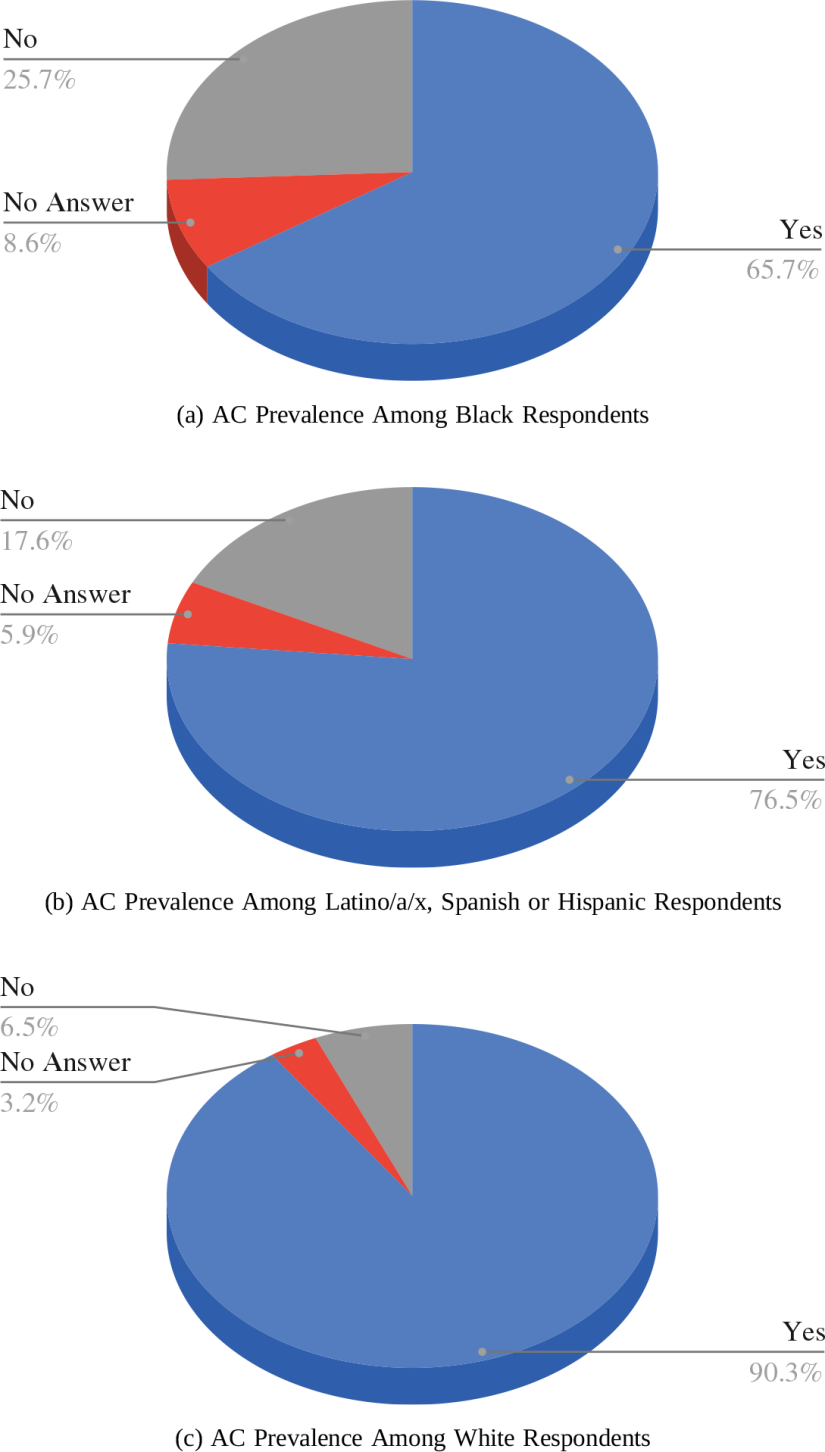
AC Prevalence by Race

**Figure 11.**
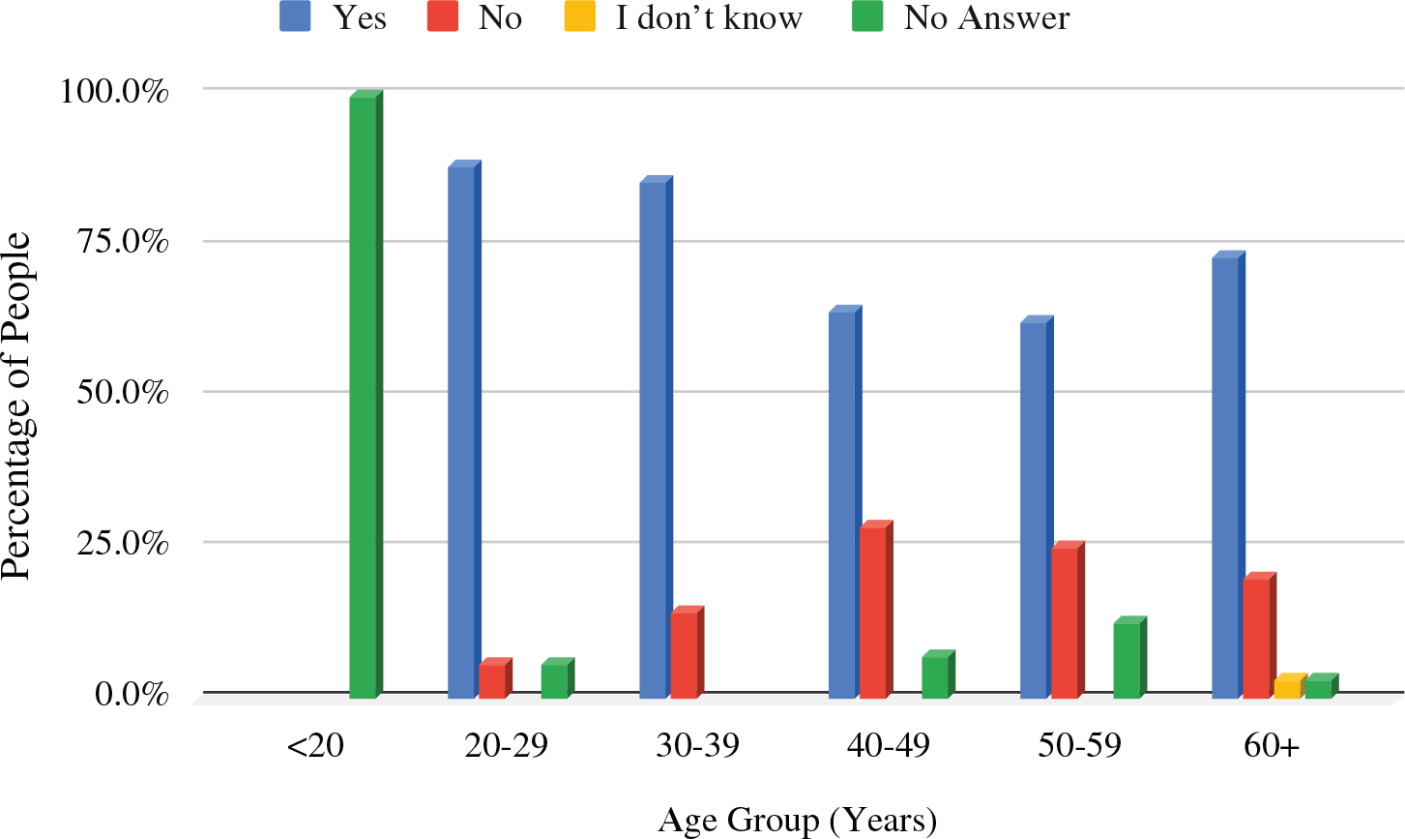
Breakdown of AC Prevalence by Age

Figure 10 illustrates a stark difference in AC prevalence between white, Latino/a/x, Black, and mixed-race respondents. AC prevalence was 90.3% for white respondents while AC prevalence for Latino/a/x respondents was 76.5%, 65.7% for Black respondents, and 60% for mixed-race respondents. AC prevalence was highest for seniors age 60 years old and above compared to other age groups (see Figure 11).

## 5. Discussion

### 5.1. Survey results

It is important to first recognize the recommendations the community groups and research organizations including WE ACT, New York City Environmental Justice Alliance (NYC-EJA), the SWIM Coalition, and the Urban Design Forum have made to improve government heat resiliency and cooling programs. Incorporating the recommendations of community organizations into the discussion of our survey results and policy review aligns with our aims to conduct community participatory research that is informed by practice-based, community knowledge. These groups can also be trusted because they are informed by the public and members who are directly impacted by COVID-19 and extreme heat. They also do not have a conflict of interest because none has large corporate donors. However, it is important to acknowledge that these organizations have their own agendas and concerns and therefore any recommendations they make should be taken with those in mind.

We also acknowledge the limitations of the survey data. The small sample size of the survey and lower response rate limit the conclusions we can make based on the results. The sample population could also be more representative of vulnerable communities in NYC. Our study primarily surveyed Harlem residents because most WE ACT members live in Northern Manhattan. The survey results may not accurately reflect the attitudes and experiences of the wider population because the participants we surveyed may be more civically engaged and aware of heat-related health issues as members of WE ACT than the average member of the public. There were also limitations to our survey methodology. Due to social distancing requirements, the survey was only administered online. Despite these limitations, we were able to make the following observations.

Our survey results reflect a larger trend in Summer 2020 of NYC residents spending much more time indoors in part due to COVID-19. People spending more time indoors may have increased their risk of heat-related illnesses because more heat-related illnesses and deaths typically occur indoors and the risk increases based on access to cooling and socioeconomic factors (Walters *et al*. 2014; Vant-Hull *et al*. 2018). Two-thirds of heat-related deaths in NYC occur at home due to issues such as lack of mobility, lack of AC access, and apartments not cooling down in the evening (Walters *et al*. 2014; Lane *et al*. 2014; Vant-Hull *et al*. 2018).

According to our survey results, more people spent their time indoors during hot days in comparison to past summers which increased the need for people to use indoor cooling mechanisms such as air conditioning. Data indicated that in past summers white respondents spent more time indoors but elsewhere besides their home compared to Latino/a/x and Black respondents spending more time indoors at home. This could potentially demonstrate where the need for indoor residential cooling mechanisms is highest. During Summer 2020 nearly everyone spent more time indoors at home due to COVID-19. The survey data shows less people went outside and accessed green space. The qualitative results of respondents’ comments show a clear trend that people visited parks less due to COVID-19 and concerns around overcrowding. Our results also showed that people who lived farther away from green space visited it less which demonstrates the importance of developing more green spaces to improve access for outdoor cooling.

### 5.2. Government policies and tools

When evaluating the government’s response to the compounding health risks, COVID-19, and extreme heat presented for vulnerable communities, it was important to contextualize policies that were already in place to highlight what further action the government could take. This section is important to understand the current heat resiliency policies in place and what was possible under the special circumstances of the pandemic. A review of government policies also provided context to analyze our survey results and allow us to do an initial evaluation of how well the government’s heat resiliency policies were implemented and how effective they were in targeting vulnerable populations.

#### 5.2.1. Health and social services programs

The City has created various health and social services programs that aim to protect heat-vulnerable communities with the most recent program being the Get Cool NYC program launched in the midst of COVD-19. Our survey results reflect a potential racial disparity in AC access, with AC prevalence highest amongst white respondents and lowest amongst Latino/a/x and Black respondents. Our results also showed more people spent hot days indoors. These two trends combined confirm the concerns expressed by community groups and City officials that inequitable AC access during the summer would create multiplicative health risks for vulnerable communities already experiencing disproportionate health impacts from COVID-19 (Brannan and Salamanca 2020; Mercado 2020; NYC-EJA 2020; Cary Institute 2020). These heightened health risks caused by the pandemic may have been what led the City to finally invest in the free AC program that advocates, such as Kizzy Charles-Guzman, the Deputy Director of the Mayor’s Office of Sustainability, had long asked for (Charles-Guzman 2020a). In turn with an increased reliance on ACs and the creation of the Get Cool NYC program, energy usage and affordability issues and the need to expand the City and State’s retrofit programs to increase energy efficiency and improve building insulation was also made more apparent. Although the City had created other heat resiliency programs and policies in the past to address health risks posed by extreme heat, the Get Cool NYC program represented a shift in the City’s willingness to commit significant funding and staff resources to address the issue on a larger scale (Charles-Guzman 2020b). The City spent $106 million on the Cool Neighborhoods NYC program, a multiyear program which encompassed many different components such as targeted tree planting and pilot community outreach programs. In comparison, all within the span of a few months, the City spent $55 million on purchasing ACs for low-income seniors and the NYS Public Service Commission spent $70 million on subsidizing utility bills for low-income residents.

Considering the ambitious timeline and goals of the Get Cool NYC program and the logistical challenges the City faced with implementation, the program was successful in providing cooling assistance to at least some New Yorkers. It will be key to do a more thorough assessment to measure how effective the program was in helping those most in need. According to community groups, the program was disorganized and did not reach everyone needed. Some seniors said outreach was conducted in a bureaucratic, inefficient manner (Fitzsimmons 2020). WE ACT said a lot of seniors were told they qualified but as of late August were still waiting for ACs (Fitzsimmons 2020). The Stanley M. Isaacs Neighborhood Center in Manhattan spent $30,000 on 56 ACs for seniors because of the program’s delays. The director of the center said the City should have worked with community groups who could have provided a list of older residents who qualified for the program (Fitzsimmons 2020). There may be data privacy issues that make it difficult to identify eligible program participants. However, coordinating outreach with community groups who have access to their own contact lists can help more easily identify eligible participants. WE ACT recommends the City expand the definition of who qualifies for the program beyond senior citizens so that others in need such as people with chronic illnesses can gain access to ACs (Mercado 2020).

Increasing access to ACs also requires providing adequate energy bill assistance to offset the prohibitive costs of using an AC. Our survey results indicate a slight increase in electricity costs which could be due to an increased use of AC because more people were indoors. In terms of AC usage, the slight increase in electricity costs aligns with the increase in time spent at home. AC prevalence by race shows a racial disparity in AC access with white respondents having a higher AC prevalence than Latino/a/x and Black respondents. This racial disparity in AC access is reinforced by previous studies (Rosenthal *et al*. 2014). However, the sample size was small enough that this trend needs further exploration. AC prevalence by age generally decreased as the age of respondents increased, apart from the trend that 60 years old and above respondents seemed to have the highest AC prevalence. This could indicate that the Get Cool NYC program has been effective in giving ACs to seniors. Community groups and past studies highlight that one of the main barriers to increasing AC access is high electricity costs associated with using an AC. Although there are programs in place to address this issue such as HEAP, the program is limited in who is eligible for the program and its funding is still mostly used for winter heating assistance rather than summer cooling assistance. The City was still forced to ask for more funding to provide low-income New Yorkers with utility bill assistance during the implementation of the Get Cool NYC program from Con Edison, the Public Service Commission and NYSERDA (Fitzsimmons 2020). WE ACT recommends New York State expand HEAP to increase AC access and reduce the economic burden of electricity use for vulnerable populations. Ms. Charles-Guzman supports the call for NYS to reform the HEAP program and says the program is a more sustainable, long-term solution to increase equitable AC access. She says it is more efficient for NYS to manage the process of distributing free ACs to vulnerable populations through already established channels like the HEAP program compared to NYC which has more limited staff capacity and funding (Charles-Guzman 2020b). Groups like WE ACT and NYC-EJA as well as government officials such as Ms. Charles-Guzman and State Senator Gustavo Rivera and Assembly member Andrew Hevesi also recommend the State expand the program to finance energy efficiency retrofits and revise the definition of eligible recipients for HEAP to promote equity and increase support to vulnerable populations that do not meet the State’s current eligibility requirements (WE ACT 2020; Charles-Guzman 2020b; Hevesi and Rivera 2020; NYC-EJA 2020). Members of Congress also supported expanding the eligibility of the national Low-Income Home Energy Assistance Program (LIHEAP) to provide energy cooling assistance during Summer 2020 due to COVID-19 (Bonamici *et al*. 2020).

With regards to other health and social services programs aimed at targeting heat-vulnerable populations, especially elderly low-income communities of color, community groups have various recommendations to improve the City’s home health aide climate training, Be a Buddy program, and the cooling center program. WE ACT recommends the City require that home health aides and cooling center staff participate in training to learn how to identify heat-related health impacts. Currently the training is done on an ad hoc, voluntary basis. WE ACT and NYC-EJA recommend the City expand and permanently fund the Be a Buddy program. NYC-EJA and the POINT CDC, which was one of the groups that implemented the pilot program, also advocate for the City to prioritize community preparedness and awareness projects such as the Be a Buddy program. The network created through the Be a Buddy program proved to be an effective way to reach out to vulnerable community members in the height of the COVID-19 pandemic during the Spring and Summer of 2020. The POINT CDC was able to identify people who needed groceries or PPE delivered and connect community members to social services through the network (Ortiz 2020). Mutual aid groups that have become very active during the pandemic also proved to be effective informal networks during the summer to check in on neighbors and share heat health safety information (Flavelle and Moran 2020). An Urban Design Forum report published in June 2020, which was written in partnership with the Mayor’s Office of Resiliency also recommended the City expand the Be a Buddy program by creating a city heat wave risk registry that would create a list of heat-vulnerable individuals and medical and emergency personnel that provide emergency response during a heat wave. The registry would be used for targeted outreach during a heat wave (Urban Design Forum 2020). Other cities around the world have introduced a heat wave directory similar to this idea. Paris, France created a heat wave directory in 2004 in response to the 2003 heat wave that is used to do targeted outreach to people who voluntarily registered to join the directory (Urban Design Forum 2020).

With respect to the City’s cooling center program, NYC-EJA and WE ACT recommend the City’s list of official cooling centers should be available all the time and be updated in real time (WE ACT 2020; NYC-EJA 2020). The City currently only publishes a list of cooling centers on its website during a heat emergency. According to a 2019 report of the City’s cooling centers during the summer of 2019, many facilities did not even know they were cooling centers, were closed or did not have working ACs or running water (Murphy 2019). A cooling center survey conducted by WE ACT in 2019 found that 88% of cooling centers in Northern Manhattan were open and functioning in Summer 2019. 27% of 44 cooling centers WE ACT assessed had signage directing people to a facility (Jessel and Solecki 2020). During Summer 2020, many cooling centers were not open or were slow to open due to the pandemic. COVID-19 forced the City to think creatively about how to provide safe public cooling centers that complied with social distancing measures. The City experimented with using public school buildings as cooling centers (Charles-Guzman 2020b; Brannan and Salamanca 2020; Culliton 2020). WE ACT recommends the City establish a more comprehensive criteria that all facilities need to meet in order to become a cooling center and improve its communication for promoting the use of cooling centers such as using more signs and implementing neighborhood specific communication strategies. They also recommend that staff at cooling centers be better informed that their facility is a cooling center and be trained how to identify heat stress in site visitors. In Spring 2020, the New York City Council Committee on Resiliency and Waterfronts cited the need to reform the City’s cooling center program given the heightened health risks low-income communities of color faced from COVID-19 and extreme heat. WE ACT AND NYC-EJA along with City Council members such as Justin Brannan support the introduction of New York City Council bill 1563–2019 which would codify cooling centers in NYC (WE ACT 2020; Brannan and Salamanca 2020). Proposed in 2019, the bill includes a set of guidelines to improve the effectiveness of the City’s cooling center program. The legislation gives the DOHMH and OEM the authority to designate the number and location of cooling centers and requires them to take into account where heat-vulnerable populations live and where they would be most likely to use centers. Currently 32% of seniors live more than half a mile away from cooling centers (Schuerman 2019). The bill also requires the DOHMH post information about the cooling centers on its website and conduct public education campaigns to increase awareness of the cooling center programs and the risks associated with heat emergencies (NYC City Council 2019). Urban Design Forum recommends the City expand the reach of its cooling centers by creating informal and pop-up cooling centers and engage communities on how to best design cooling centers that are accessible and meet the community’s needs.

#### 5.2.2. Built environment

Along with addressing the immediate health risks posed by extreme heat and COVID-19, it is also important the City continue to implement programs and policies that aim to address and reverse the pervasive infrastructure inequities that exacerbate these risks. The City’s Cool Roofs program is a good example of one of its earlier programs that attempted to address heat and building energy usage issues. Urban Design Forum recommends the City continue to invest in cool roofs for existing buildings and pilot new technologies to increase the effectiveness of cool roofs such as high-reflectance paint. Cool walls, walls painted with reflective paint that reflect sun and heat back into space, are another design solution urban planners have proposed for reducing building heat absorption that may be more effective in reducing UHI effects since it involves more heat being reflected off a building than just the roof (Urban Design Forum 2020). Urban Design Forum recommends the City pass a zoning code that requires the use of cool walls similar to the existing codes for cool roofs.

To address the issue of increased energy usage putting pressure on peak energy demand during a heat emergency, City Council members as well as community groups like WE ACT recommend the City coordinate emergency planning strategies to prevent power outages and promote safety (WE ACT 2020; Brannan and Salamanca 2020). WE ACT also recommends the City preemptively set maximum temperatures for larger buildings to reduce energy loads and improve the delivery of portable generators. The Urban Design Forum and WE ACT recommend NYC City Council pass legislation that establishes a maximum indoor temperature threshold for apartment buildings and facilities that house heat-vulnerable populations (Urban Design Forum 2020; WE ACT 2020). This legislation would mirror existing NYC laws that set minimum indoor temperatures of 68°F (20°C) for apartments when outside temperatures fall below 55°F (~ 13°C) (WE ACT 2020; NYC-EJA 2020). The City’s OneNYC sustainability plan assessed the feasibility of a maximum allowable indoor temperature for residential facilities and other facilities where vulnerable communities live. However, after conducting interviews with different stakeholders about a maximum indoor temperature, they determined it would pose significant challenges and required further examination (MOR 2017). Urban Design Forum recommends the City expand Local Law 84 to require benchmarking for building temperature and expand Local Law 86 to include a maximum indoor temperature. They recommend expanding Local Law 87 to require energy efficiency audits for smaller multi-family buildings.

Urban Design Forum also recommends incorporating extreme heat as a “life safety issue” in building codes and specify shade requirements for zoning ordinances. They recommend the City create heat-vulnerable neighborhood zoning policy that requires new projects to consider the impacts of development projects on an area’s micro-climate and how it can reduce the area’s HVI during the environmental review process (Urban Design Forum 2020).

Poor quality of housing conditions also contributes significantly to heat vulnerability (Rosenthal *et al*. 2014). WE ACT recommends NYCHA develop an emergency plan for extreme heat due to the high number of heat-vulnerable individuals that live in NYCHA housing. According to NYC-EJA’s Climate Justice Agenda 2020 report, a majority of NYCHA’s residents are located in heat-vulnerable neighborhoods (NYC-EJA 2020). They call on the City to require NYCHA to implement the findings from its Sheltering Seniors from Extreme Heat study to reduce heat retention in its buildings. NYCHA began two pilot projects in 2019 to install smart air conditioners in the Meltzer Tower in Lower Manhattan and a heat pump pilot at the Fort Independence Houses in the North Bronx (NYC-EJA 2020). However, both of these projects are in neighborhoods that have lower heat vulnerability scores and thus lower heat-health risks (NYC-EJA 2020). NYCHA should prioritize expanding these programs in the city’s most heat-vulnerable neighborhoods such as Northern Manhattan, the South Bronx, and Central Brooklyn. The NYC-EJA member group Community Voices Heard also recommends NYCHA allocate $450 million of its state funding to repairing broken HVAC systems. Many of NYCHA’s Senior Centers that have served as cooling centers for heat-vulnerable communities have persistent problems with broken HVAC systems, prohibiting these facilities from providing relief during an extreme heat event (NYC-EJA 2020). Because NYCHA buildings do not allow for central air conditioning and thus rely on window AC units, heat generated from AC units that is released outside can contribute to increased outdoor temperatures (Ohashi *et al*. 2007). Heat emitted from AC units can account for up to 15% of heat in a heat wave and may be a significant contributor to the UHI effect (Sailor 2011; Olivo *et al*. 2017). To address this issue, the Urban Design Forum 2020 report recommends a NYCHA pilot study be conducted to measure heat exhaust from ACs and determine effective methods for reducing heat capture from AC units such as increased building insulation (Urban Design Forum 2020).

#### 5.2.3. Green space

Our results indicate that NYC residents tended to avoid green spaces due to concerns around overcrowding and social distancing as well as because some lived too far away from green spaces. This confirms the call by community groups for the City to provide more equitable access to green space. The inequitable access to green space has become more pronounced during the COVID-19 pandemic with low-income communities of color having less ability to practice safe social distancing and benefit from the physical and mental health effects provided by green spaces due to a lack of green space in their area (Lopez *et al*. 2020). A study that ranked park systems in the 100 most populous cities found parks that serve a majority non-white population are half as large and five times more crowded than parks that serve a majority white population (Trust for Public Land 2020). The study also found parks serving low-income households are four times smaller than parks that serve majority high-income households.

Green space such as street trees, parks, urban forests, and green roofs is a key solution to reducing air temperature and providing safe outdoor cooling corridors for residents, which has been especially crucial during the COVID-19 pandemic when cooling centers had challenges being open during the summer due to social distancing concerns (Mercado 2020). The City should work with local communities to ensure that expansion of green space does not lead to green gentrification, a common outcome of increasing green space access (Anguelovski *et al*. 2019). WE ACT recommends the City equitably distribute green roofs and solar roofs prioritizing development in environmental justice and heat-vulnerable communities as part of the Climate Mobilization Act’s Local Law 92 and 94. They also call for green design techniques including installing shading over pedestrian areas, planting more vegetation, and green spaces in high heat-vulnerable areas and increasing renewable energy production to promote natural cooling and reduce NYC’s carbon emissions. Urban Design Forum and the SWIM Coalition also support equitable distribution of green roofs. The SWIM coalition recommends the NY State Green Roof Tax Abatement policy be reformed to increase the abatement amount for green roof installations in non-priority areas from the current $5.23 to $15 per square foot everywhere with a higher amount in priority areas. Currently the $15 per square foot amount only applies to high priority areas.

NYC-EJA calls for the City to expand the Cool Neighborhoods NYC’s commitment to planting street trees in neighborhoods which have the most need, given the importance of street trees and urban forests in reducing the UHI effect and air pollution. They also advocate for the City to renew the MillionTreesNYC program to increase the urban tree canopy coverage and ensure the long-term maintenance of the city’s urban forests (NYC-EJA 2020). Currently the City’s OneNYC 2050 sustainability plan does not commit to further investment in planting trees in heat-vulnerable areas.

As we have discussed, there are various State and City policies that aim to reduce heat vulnerability. Some of these policies have been more effective at targeting those most vulnerable to the health impacts of extreme heat. The Get Cool NYC program, Cool Neighborhoods NYC program (including the Be a Buddy program and Street Tree program), as well as the State’s new Green Roof Tax Abatement policy and the HEAP program are more targeted initiatives that prioritize NYC’s most heat-vulnerable communities.

#### 5.2.4. Heat emergency response and communication

The DOHMH does outreach to the media and health care providers about heat-health effects to prevent heat associated morbidity and mortality that can occur on heat alert days as well as throughout the summer on non-heat alert days (Rosenthal and Brechwald 2013). Many groups call on the City to improve its response and communication strategies during a heat emergency. WE ACT recommends the City implement and expand channels of communication with vulnerable populations to increase awareness of extreme heat impacts. They recommend the City develop a relationship between OEM and local television and radio stations and require the announcement of heat emergencies through the emergency broadcast system. Urban Design Forum recommends heat waves be named similar to how hurricanes are named to increase awareness of the emergency.

WE ACT and NYC-EJA also support the New York City Council Law 1960–2020 passed on August 28, 2020 that requires the City to submit their summer heat plan by May 15 each year (Salamanca *et al*. 2020). The introduction and enactment of this law may have been accelerated in part due to the urgency and multiplicative health risks the COVID-19 pandemic presented. As part of the annual heat plan the City is now required to submit, WE ACT asks that the City develop a specific action plan to protect vulnerable populations during extreme heat events. They also ask the City to collaborate with heat-vulnerable communities in participatory visioning processes to develop plans for resilience to extreme heat. Urban Design Forum also recommends the City increase transit access during a heat wave emergency by subsidizing or waiving fees to seniors using reducedfare MetroCards or provide door-to-door service to cooling centers (Urban Design Forum 2020).

### 5.3. NYC heat vulnerability data

In regards to overall recommendations for the City’s heat vulnerability data, WE ACT recommends the City increase the collection of heat-related health data, analyze cumulative impacts, and share the findings with the City’s Environmental Justice Advisory Board. According to NYC-EJA and City Council Member Justin Brannan, “it is likely NYC is significantly underestimating the actual number of heat-related mortalities because deaths from other illnesses that may have been exacerbated by an extreme heat event are not counted” (NYC-EJA 2020; Brannan and Salamanca 2020). The DOHMH tracks heat-related illnesses and deaths by monitoring and analyzing data from hospital emergency department visits and Emergency Medical Service calls (Rosenthal and Brechwald 2013). They also analyze data on heat-stroke deaths during and following a heat event provided to the DOHMH by the Office of the Chief Medical Examiner. DOHMH has not updated the heat morbidity data published on its environmental health data portal since 2017 (Walters *et al*. 2014; NYC City Council 2020). NYC-EJA calls on the City to make anonymized daily-level mortality data available to the public for researchers to create models for predicting heat-related deaths with more accurate data to be able to sufficiently understand and address the issue (NYC-EJA 2018). NYC-EJA also supports City Council Speaker Cory Johnson’s call for legislation requiring DOHMH to re-evaluate its metrics for counting heat-related deaths and illnesses (NYC-EJA 2020). Urban Design Forum recommends the City establish neighborhood specific heat vulnerability designations and refine the definition of heat-vulnerable communities based on more localized and human-scale indicators to inform policy decisions and resource allocations. In addition to the City’s existing HVI indicators, they recommend adding the following data indicators: ground and building-reported temperature instead of using recorded temperatures at Central Park or airports, detailed analysis of the built environment in high heat-vulnerable neighborhoods, analysis of a neighborhood’s specific vulnerability indicators including income, race, social cohesion (Urban Design Forum 2020). The City could use existing already installed air quality monitors located around the city to collect temperature data. The recently passed NY City Council Law 1945–2020, also supported by WE ACT and NYC-EJA, which requires the DOHMH to annually report on neighborhood heat vulnerability and the number of heat-related deaths, will further improve the City’s collection of heat vulnerability data.

#### 5.3.1. NYC HVI data tools

The major limitation with the City’s HVI maps is that they do not show heat vulnerability on a more granular street level view which would help increase the specificity and effectiveness of the City’s targeted heat-related health interventions and better inform people what their home’s heat vulnerability is. The maps previously showed data by community district, which is not a recognizable geographic indicator for most New Yorkers. The City’s most recent HVI map (see Figure 1) shows data by neighborhood tabulation, which is an improvement. Another limitation of the maps is that they only use land surface temperature data compared to other heat indices that include humidity and air temperature. Humidity is potentially a major contributor to causing heat-related health issues (Sobolewski *et al*. 2019). UHI effect is often measured using air temperature because that is what people feel and affects human thermal comfort (van Hove *et al*. 2015; Li *et al*. 2019). The study that the City’s main HVI map is based on only uses land surface temperature data from a series of heat wave events at certain hours of the day. This can lead to the HVI tool underestimating heat-related mortalities because heat-related health impacts can occur outside of heat wave events (Murage *et al*. 2017). The NYC Office of Emergency Management activates a citywide heat emergency plan and declares a Heat Advisory when the National Weather Service predicts a 24- to 48-hour forecast maximum heat index of 95°F (35°C) or above for two days or 100°F (~ 38°C) or above for one day (Rosenthal and Brechwald 2013). With extreme heat events occurring more frequently and lasting longer due to climate change, basing the citywide heat warning system on such a narrow index may not be as accurate of a metric in preventing heat illnesses. The City should reassess this heat index and consider adding a humidity layer and more varied temperature data to calculate a more accurate HVI.

The HVI website also educates the user about the issue of extreme heat and the factors that contribute to why heat vulnerability is higher and lower in certain parts of the city citing environmental and socioeconomic factors including surface temperature, proximity to green space, access to home air conditioning, race and income (see Figures 1 and 2). The City can improve its HVI mapping tools by suggesting actions people can take to reduce their heat vulnerability and share resources and links to the City’s other tools that include information about the City’s cooling programs and amenities such as cooling centers and the Cool It! NYC map that shows where sprinklers and fire hydrants are (see Figure 3). In regards to the Cool It! NYC Map, it may be helpful to add an option to only view the cooling amenities that are available every day to exclude all the “only heat emergency” amenities and increase clarity.

## 6. Conclusion and Recommendations

Our survey results and policy review provide community groups, researchers, and policy-makers an initial evaluation of how effective the government’s response was in addressing compounding health risks created by COVID-19 and extreme heat and how to prepare for extreme cascading events similar to these in the future. Our survey results reinforce the trend observed in previous literature that there is a racial disparity in access to cooling mechanisms such as AC and green space. Our survey results that showed people spent more time indoors compared to past summers also align with the concerns expressed by community groups and City officials around the urgent need to provide indoor cooling to vulnerable populations due to social distancing measures during the pandemic. These findings highlight how the COVID-19 pandemic heightened the need to accelerate efforts to improve targeted interventions like the City’s free AC and cooling center programs, HVAC retrofitting through HEAP, and green space expansion to high heat-vulnerable areas. Our findings also highlight the need to develop stronger environmental justice community networks and feedback mechanisms to hear from and check in on impacted residents. Our review also revealed that many of these community groups like WE ACT have concrete and useful recommendations for improving the growing list of programs at the City and State level designed to address heat vulnerability and some of the factors that contribute to its unequal distribution across NYC. Requiring the City to produce a yearly strategy to address extreme heat might also be one way to introduce more data-driven accountability into the City’s interventions and strive for continued more inclusive, targeted, and effective planning and program implementation.

To improve the usefulness of the City’s data tools, we recommend the City integrate all the heat-related data it has into one centralized location that shows mapping of heat vulnerability data, locations of hospitals, tree canopy cover, and locations of green spaces and cooling amenities. We also recommend this centralized website share information about how people can take action to reduce their heat vulnerability and link to more information about City and State cooling programs such as the public cooling centers, HEAP utility bill assistance, and the Get Cool NYC free AC program. This centralized website should also be a user-friendly tool that provides more neighborhood-specific and street-level information. In line with what community groups have asked for, the City should also update the heat-related mortality data it uses and expand the indicators it uses to measure heat-related mortality and heat vulnerability. The City should also reassess what variables it uses to create a heat index. This will help improve the accuracy and specificity of the HVI tool and allow the City and researchers to make program and funding decisions that better target heat-vulnerable communities. As groups have pointed out, heat-related health impacts could be underestimated, leaving many people to fall through the cracks in benefiting from the City’s programs.

Future research regarding heat vulnerability needs to bring in more targeted community feedback. The City can partner with community groups who are already doing air quality monitoring to collect temperature data using micro air quality monitors. There should also be more regular surveying of communities to understand their needs and to evaluate how the City’s programs are performing in terms of meeting their needs and keeping people safe during heat emergencies. We recommend conducting surveys in other heat-vulnerable areas of NYC such as the South Bronx to determine if trends are similar across the city. A systematic and comprehensive review of the City’s HVI tools and cooling programs is needed in order to determine how effective they are in reducing heat vulnerability and targeting NYC’s most vulnerable communities. We also recommend annual systematic surveys of the same areas be conducted to understand and evaluate the effectiveness of cooling initiatives over time.

Advocates have long been asking the City and NYS to provide more interventions and funding to support community preparedness and address AC access and infrastructure issues that contribute to higher heat vulnerability for low-income communities of color. The creation of the Get Cool NYC program demonstrates the City is beginning to prioritize heat vulnerability issues in a more substantive way. However, it is still unclear how impactful the intervention was and if it will become a permanent program. Community groups and City officials recommend NYS reform the HEAP program to make it easier for vulnerable populations to qualify for free ACs and utility bill assistance. More research should be conducted to evaluate how effective the City and State’s interventions have been overall in reducing heat vulnerability and related health impacts. With current funding streams running out for interventions such as the City’s Cool Neighborhoods NYC Street Tree planting and Be a Buddy programs and given the current period of budget scarcity the City is in, the future of these health equity-focused programs is uncertain despite the need for these types of interventions being even greater, with COVID-19 only heightening heat-related health risks for vulnerable communities. Policies such as the proposed City Council Bill 1563–2019 that codifies the City’s cooling center program and recently enacted City Council Law 1960–2020 that requires the City to submit a heat plan annually need to be implemented with a core equity focus in order to allocate permanent resources and institutionalize these crucial, life-saving programs.

## Figures and Tables

**Table 1.** Government Agency Terms

NYC Agency Key:	NY State Agency Key:

MOR = Mayor’s Office of Resiliency	OTDA = Office of Temporary and Disability Assistance
DOHMH = Department of Health and Mental Hygiene	HCR = Housing and Community Renewal
MOS = Mayor’s Office of Sustainability	NYSERDA = New York State Energy Research and Development Authority
NYCPARKS = NYC Department of Parks and Recreation	NYSOA = NYS Office of the Aging
NYCHA = New York City Housing Authority DSBS = Department of Small Business Services DCAS = Department of City Administrative Services	NYSDOH = NYS Department of Health
DOB = Department of Buildings DOF = Department of Finance OEM = Office of Emergency Management DOA = Department of Aging	

**Table 2.** NYC and NY State Cooling Programs

Program or Policy		Year Started	Agencies Involved	Description

Cool Roofs NYC		2009	MOS, DSBS, MOR	NYC program created in 2009 as part of the City’s goal to reduce its greenhouse emissions by 80% by 2050. By painting a roof with a reflective coating, less heat is absorbed by the building, which reduces the indoor temperatures and the need for AC.
Home Energy Assistance program (HEAP)		2013	OTDA, HCR, NYSERDA, NYSOA, NYSDOH	State program created in 2013 that helps low-income New Yorkers pay for the cost of heating and cooling their homes.
Cool Neighborhoods NYC		2017	MOS, MOR, DOHMH, NYCPARKS	NYC program created in 2017 that established the City’s first comprehensive heat resiliency plan that focuses on reducing heat-related health impacts and deaths by lowering temperatures in heat-vulnerable neighborhoods and strengthening social networks.
Sub-Program	Home Health Aide Training	2017	DOHMH, MOR	NYC initiative created as part of Cool Neighborhoods NYC that provides climate risk training for home health aides.
Sub-Program	Street-Tree Planting	2017	MOR, NYCPARKS, DOHMH	NYC street-tree planting program that targeted planting trees in heat-vulnerable areas identified based on the city’s HVI.
Sub-Program	Be a Buddy Program	2018	MOR, DOHMH	NYC program created in 2018 as part of the Cool Neighborhoods NYC program to promote community cohesion and collaboration with community groups to develop and test strategies for protecting at risk New Yorkers from the health impacts of extreme heat in heat-vulnerable areas (NYC Office of the Mayor).
Sub-Program	Cool It! NYC	2017	NYCPARKS, NYCHA	NYC program created as part of the Cool Neighborhoods NYC program to increase the amount of cooling amenities available to the public.
Cooling Centers		2017	MOR, DOHMH, DOA	NYC opens designated cooling centers at locations such as community centers, senior centers, public libraries during a heat emergency to provide a safe and cool place for heat-vulnerable community members to go.
Introduction 1563–2019 (Proposed)		2019	DOHMH, OEM	Introduction 1563 is a NYC City Council bill proposed in May 2019 which would codify the City’s cooling center program and includes a set of guidelines to improve the effectiveness of the program.
Get Cool NYC		2020	MOS, MOR, DOHMH, DCAS, DOA, NYCHA, OEM	NYC program created in May 2020 that provides free air conditioners to low-income seniors who are enrolled in city benefit programs (NYC 311 Portal).
Green Roofs Tax Abatement Law		2019	DOB, DOF, NYCPARKS	State law that provides a one-time property tax abatement of $5.23 per square foot of green roof space for properties that have green roofs. The benefit is capped at whichever is less: $100,000 or the amount of property taxes due for the building that tax year (DOF).
	Local Law 92 & 94	2019	DOB, DCAS, NYCPARKS	NYC laws that require all new buildings or existing buildings that need to replace the whole roof to build either green roofs or solar panels (DOB).
Climate Mobilization Act	Local Law 97	2019	DOB, DCAS, MOS	NYC law that requires buildings larger than 25,000 square feet (50,000 residential and commercial buildings in NYC) to reduce greenhouse gas emissions by 80% by 2050 (Building Energy Exchange).
	Local Law 98	2019	DOB, MOS, DCAS	NYC law that requires DOB to include wind energy in its use of renewable energy technologies (Building Energy Exchange).
Introduction 1960–2020 (Enacted)		2020	DOHMH	Introduction 1960–2020 is a NYC law enacted in 2020 which requires the City to submit their summer heat plan by May 15th each year.
Introduction 1945–2020 (Enacted)		2020	DOHMH	Introduction 1945–2020 is a NYC law enacted in 2020 which requires the DOHMH to annually report on neighborhood heat vulnerability and the number of heat-related deaths.

**Table 3.** NYC Data Tools

Data Tool	Agencies Involved	Description

Heat Vulnerability Index (HVI)	DOHMH, MOR	The HVI measures how vulnerable neighborhoods are to heat on a scale of 1–5 based on environmental variables including daytime summer surface temperature data, percent of green space land surface temperature, and social variables including poverty measured by the percent of people living below the federal poverty level, percent of non-Latino/a/x Black residents data, and access to air conditioning data.
HVI Hospital/Green Space Map	DOHMH	HVI map that allows users to see the heat vulnerability of a neighborhood and what hospitals and green spaces are located in this neighborhood.
HVI Neighborhood Map	DOHMH	HVI map that is broken down by neighborhood and allows users to look up their heat vulnerability by neighborhood.
Cool It! NYC Map	NYCPARKS	Map that shows cooling amenities people can access to cool down during a heat emergency.
Street Trees Map	NYCPARKS	Map that shows the number and type of street trees across NYC.

## Footnotes

1 The City’s Heat Vulnerability Index (HVI) shows neighborhoods whose residents are more at risk for dying during and immediately following extreme heat (DOHMH 2020). Neighborhoods are scored from 1 (lowest risk) to 5 (highest risk) (DOHMH 2020).

2 IRB AAAT1940.
